# A generalized exponential-type estimator for population mean using auxiliary attributes

**DOI:** 10.1371/journal.pone.0246947

**Published:** 2021-05-13

**Authors:** Sohail Ahmad, Muhammad Arslan, Aamna Khan, Javid Shabbir

**Affiliations:** 1 Department of Statistics, Quaid-i-Azam University, Islamabad, Pakistan; 2 School of Statistics, Shanxi University of Finance and Economics, Taiyuan, China; 3 Department of Statistics, Bahauddin Zakariya University, Multan, Pakistan; 4 Department of Statistics, Quaid-i-Azam University, Islamabad, Pakistan; Tongii University, CHINA

## Abstract

In this paper, we propose a generalized class of exponential type estimators for estimating the finite population mean using two auxiliary attributes under simple random sampling and stratified random sampling. The bias and mean squared error (MSE) of the proposed class of estimators are derived up to first order of approximation. Both empirical study and theoretical comparisons are discussed. Four populations are used to support the theoretical findings. It is observed that the proposed class of estimators perform better as compared to all other considered estimator in simple and stratified random sampling.

## Introduction

In survey sampling, we generally use the auxiliary information to increase precision of the estimators by taking the advantages of correlation between the study variable *y* and the auxiliary variable *x*. When the relationship between *y* and *x* is positive, then the ratio estimator gives efficient results, and when the relationship is negative, then the product estimator performs better. However under different setup, the auxiliary attributes have been studied by different authors. In some situations, auxiliary information can be quantified in the form of auxiliary proportions to get better precision. For this reason, several authors have used one or more auxiliary proportions at the estimation stage to increase the efficiency of the estimators. The ratio, product and regression methods of estimation are the good examples in this context. These methods of estimation are more efficient than the usual mean per unit estimator under certain conditions. Different estimators have been suggested or modified by many authors when used mixed types estimators i.e using regression and ratio type estimators or exponential type estimator. These mixed estimators outperformed than the individual estimators. For this reason, several authors have used the auxiliary variables and auxiliary attributes at estimation stage to increase the efficiency of the estimators. For example, the expansiveness of a tree can be used as a key auxiliary variable while estimating the ordinary stature of trees in a forest and moreover the sort of a dairy creatures is a significant auxiliary characteristic while estimating typical milk yield. Additionally, to estimate the mean time-based compensations earned by the individuals, the auxiliary attribute can be utilized in type of the education and martial status etc.

Wynn [1] proposed an unbiased ratio estimator using the auxiliary characters in the form of known population proportion of the auxiliary variable when the population is divided into two classes. Singh et al. [2] suggested ratio of proportion utilizing the auxiliary variables for further investigation. Naik and Gupta [3] introduced the idea of point bi-serial correlation co-efficient. Using this idea, many authors have used the information on the auxiliary attribute for improving the precision of the estimators. Solanki and Singh [4] suggested a class of estimators for population mean of the study variable using known population proportion of the auxiliary attribute. The suggested class of estimators is more general and includes the usual unbiased sample mean estimator. Expressions of bias and mean square error (MSE) are obtained under large sample approximation. Malik and Singh [5] introduced the exponential type estimator using two auxiliary attributes. Zeng et al. [6] proposed Tobit model for the analysis of crash rates by injury severity for both correlation across injury severity and unobserved heterogeneity across road-segment observations are accommodated. The Tobit model is compared multivariate parameters in the context of Bayesian. In anaother study, Zeng et al. [7] researches multivariate spatio-transient examination for characterizing territory wide accident rates by injury seriousness. Likewise, Zeng et al. [8] investigated the connection between zone-level daytime and evening time crash frequencies and different elements identified with traffic, organization, and land use, including VHT, normal speed, street thickness, crossing point thickness, network example, and land use design.

Consider a finite population *U* = {*U*_1_, *U*_2_,…,*U*_*N*_} of size *N*. We draw a sample of size *n* by SRSWOR from a population *U*. Let *y*_*i*_ be the study variable and *ϕ*_*i*_ be the characteristics of the auxiliary attribute i.e *ϕ*_*i*_ = 1 if the *i*^th^ unit possess attribute and *ϕ*_*i*_ = 0, otherwise. Let $A=\sum_{i=1}^{N}\phi_{i}$ be the total number of units in the population possessing attribute *ϕ*_*i*_ and $a=\sum_{i=1}^{n}\phi_{i}$ be the total number of units in the sample possessing attribute *ϕ*_*i*_. Let *P* = (*A*/*N*) be the proportion of units in the population and *p* = (*a*/*n*) be the proportion of units in the sample. Let $\bar{Y}=\frac{\sum_{i=1}^{N}y_{i}}{N}$ and $\bar{y}=\frac{\sum_{i=1}^{n}y_{i}}{n}$ be the population mean and the sample mean respectively. Let *P*_1_ and *P*_2_ be the population proportion of auxiliary attributes and *p*_1_ and *p*_2_ be the sample proportion of auxiliary attributes. Let $S_{y}^{2}=\sum_{i=1}^{N}\frac{(y_{i}-\bar{Y}{)}^{2}}{N-1}$ be the population variance of the study variable *y*. Let $S_{p_{1}}^{2}=\sum_{i=1}^{N}\frac{(p_{1}-P_{1}{)}^{2}}{N-1}$ and $S_{p_{2}}^{2}=\sum_{i=1}^{N}\frac{(p_{2}-P_{2}{)}^{2}}{N-1}$ respectively be the population variance of the auxiliary attributes *p*_1_ and *p*_2_. Let $C_{y}=\frac{S_{y}}{\bar{Y}}$ respectively be the co-efficient of variation of the study variable *y*. Let $C_{p_{1}}=\frac{S_{p_{1}}}{P_{1}}$ and $C_{p_{2}}=\frac{S_{p_{2}}}{P_{2}}$ be the co-efficients of variation of the auxiliary attributes *p*_1_ and *p*_2_. Let $S_{yp_{1}}^{2}=\sum_{i=1}^{N}\frac{\left(y_{i}{-}_{Y})(p_{1}-P_{1}\right)}{N-1}$ be the population covariance between the study variable *y* and the auxiliary attribute *p*_1_ and $S_{yp_{2}}^{2}=\sum_{i=1}^{N}\frac{\left(y_{i}{-}_{Y})(p_{2}-P_{2}\right)}{N-1}$ be the population covariance between the study variable *y* and the auxiliary attribute *p*_2_. Let $S_{p_{1}p_{2}}^{2}=\sum_{i=1}^{N}\frac{\left(p_{1}-P_{1})(p_{2}-P_{2}\right)}{N-1}$ be the population covariance between the auxiliary attributes *p*_1_ and *p*_2_. Let $\rho_{yp_{1}}=\frac{S_{yp_{1}}}{S_{y}S_{p_{1}}}$ be the population point bi-serial correlation co-efficient between the study variable *y* and the auxiliary attribute *p*_1_. Let $\rho_{yp_{2}}=\frac{S_{yp_{2}}}{S_{y}S_{p_{2}}}$ be the population point bi-serial correlation co-efficient between the study variable *y* and the auxiliary attribute *p*_2_. Let $\rho_{p_{1}p_{2}}=\frac{S_{p_{1}p_{2}}}{S_{p_{1}}S_{p_{2}}}$ be the phi-correlation coefficient between the auxiliary attributes *p*_1_ and *p*_2_. Let $e_{0}=\frac{\bar{y}-\bar{Y}}{\bar{Y}},\;e_{1}=\frac{p_{1}-P_{1}}{P_{1}}\;and\;e_{2}=\frac{p_{2}-P_{2}}{P_{2}}$ be the error terms such that *E*(*e*_*i*_) = 0, (*i* = 0,1,2), $E(e_{0}^{2})=\lambda C_{y}^{2},\;E(e_{1}^{2})=\lambda C_{p_{1}}^{2},\;E(e_{2}^{2})=\lambda C_{p_{2}}^{2},\;E(e_{0}e_{1})=\lambda \rho_{yp_{1}}C_{y}C_{p_{1}},E(e_{0}e_{2})=\lambda \rho_{yp_{2}}C_{y}C_{p_{2}}\;and\;E(e_{1}e_{2})=\lambda \rho_{p_{1}p_{2}}C_{p_{1}}C_{p_{2}}$, where $\lambda =(\frac{1}{n}-\frac{1}{N})$ and $f=\frac{n}{N}$.

## Existing estimators in simple random sampling

We discuss the following estimators available in literature.

**1.** The usual mean per unit estimator in simple random sampling is:

$${\bar{y}}_{0}=\bar{y}$$ (1)

The MSE or variance of ${\bar{y}}_{0}$, is given by:

$$Var({\bar{y}}_{0})=\lambda {\bar{Y}}^{2}C_{y}^{2}$$ (2)

**2.** The ordinary ratio type estimator, is given by:

$${\bar{y}}_{R}=\bar{y}\left(\frac{P_{1}}{p_{1}}\right)$$ (3)

**3.** The usual product type estimator, is given by:

$${\bar{y}}_{P}=\bar{y}\left(\frac{p_{1}}{P_{1}}\right)$$ (4)

It is well known that the ${\bar{y}}_{R}$ and ${\bar{y}}_{P}$ are more precise than usual mean estimator ${\bar{y}}_{0}$ when $\rho_{yp_{1}}>\frac{1}{2}\frac{C_{p_{1}}}{C_{y}}$ and $\rho_{yp_{1}}<-\frac{1}{2}\frac{C_{p_{1}}}{C_{y}}$ respectively.

The bias and MSE of ${\bar{y}}_{R}$, are given by:

$$Bias({\bar{y}}_{R})≅\bar{Y}\lambda [C_{p_{1}}^{2}-\rho_{yp_{1}}C_{y}C_{p_{1}}],$$ (5)

and

$$MSE({\bar{y}}_{R})≅{\bar{Y}}^{2}\lambda [C_{y}^{2}+C_{p_{1}}^{2}-2\rho_{yp_{1}}C_{y}C_{p_{1}}].$$ (6)

Similarly the bias and MSE of ${\bar{y}}_{P}$, are given by:

$$Bias({\bar{y}}_{P})≅\bar{Y}\lambda [\rho_{yp_{1}}C_{y}C_{p_{1}}],$$ (7)

and

$$MSE({\bar{y}}_{P})≅{\bar{Y}}^{2}\lambda [C_{y}^{2}+C_{p_{1}}^{2}+2\rho_{yp_{1}}C_{y}C_{p_{1}}].$$ (8)

**4.** Bahl and Tuteja [9] proposed ratio and product type estimators for estimating finite population mean using information on single auxiliary attribute.

$${\bar{y}}_{exp(R)}=\bar{y}exp[\frac{P_{1}-p_{1}}{P_{1}+p_{1}}],$$ (9)

and

$${\bar{y}}_{exp(P)}=\bar{y}exp[\frac{p_{1}-P_{1}}{p_{1}+P_{1}}].$$ (10)

The bias and MSE of ${\bar{y}}_{exp(R)}$, are given by:

$$Bias({\bar{y}}_{exp(R)})≅\bar{Y}\lambda [\frac{3}{8}C_{p_{1}}^{2}-\frac{1}{2}\rho_{yp_{1}}C_{y}C_{p_{1}}],$$ (11)

and

$$MSE({\bar{y}}_{exp(R)})≅{\bar{Y}}^{2}\lambda [C_{y}^{2}+\frac{1}{4}C_{p_{1}}^{2}-\rho_{yp_{1}}C_{y}C_{p_{1}}].$$ (12)

Similarly the bias and MSE of ${\bar{y}}_{exp(P)}$, are given by:

$$Bias({\bar{y}}_{exp(P)})=\frac{1}{2}\bar{Y}\lambda [\rho_{yp_{1}}C_{y}C_{p_{1}}-\frac{1}{4}C_{p_{1}}^{2}],$$ (13)

and

$$MSE({\bar{y}}_{exp(P)})={\bar{Y}}^{2}\lambda [C_{y}^{2}+\frac{1}{4}C_{p_{1}}^{2}+\rho_{yp_{1}}C_{y}C_{p_{1}}].$$ (14)

**5.** Kumar and Bhougal [10] proposed an exponential ratio-product type estimator, is given by:

$${\bar{y}}_{KB(RP)}=\bar{y}[\alpha exp(\frac{P_{1}-p_{1}}{P_{1}+p_{1}})+(1-\alpha )exp(\frac{p_{1}-P_{1}}{p_{1}+P_{1}})]$$ (15)

where *α* is unknown constant.

The bias and minimum MSE of ${\bar{y}}_{KB(RP)}$, are given by:

$$Bias({\bar{y}}_{KB(RP)})≅\bar{Y}\lambda [\frac{1}{8}(4\alpha -1)C_{p_{1}}^{2}-(\alpha -\frac{1}{2})\rho_{yp_{1}}C_{y}C_{p_{1}}]$$ (16)

and

$$MSE({\bar{y}}_{KB(RP)}{)}_{min}≅\lambda {\bar{Y}}^{2}C_{y}^{2}[1-\rho_{yp_{1}}^{2}].$$ (17)

The optimum value of *α*, is given by:

$$\alpha_{opt}≅\frac{1}{2}+\rho_{yp_{1}}\frac{C_{y}}{C_{p_{2}}}.$$ (18)

**6.** Singh and Kumar [11] suggested double ratio and product type estimators, are given by:

$${\bar{y}}_{SK(DR)}=\bar{y}(\frac{P_{1}}{p_{1}})(\frac{P_{2}}{p_{2}}),$$ (19)

and

$${\bar{y}}_{SK(DP)}=\bar{y}(\frac{p_{1}}{P_{1}})(\frac{p_{2}}{P_{2}}).$$ (20)

The bias and MSE of ${\bar{y}}_{SK(DR)}$, are given by:

$$Bias({\bar{y}}_{SK(DR)})≅\bar{Y}\lambda [C_{p_{1}}^{2}+C_{p_{2}}^{2}+\rho_{p_{1}p_{2}}C_{p_{1}}C_{p_{2}}-\rho_{yp_{1}}C_{y}C_{p_{1}}-\rho_{yp_{2}}C_{y}C_{p_{2}}],$$ (21)

and

$$MSE({\bar{y}}_{SK(DR)})≅{\bar{Y}}^{2}\lambda [C_{y}^{2}+C_{p_{1}}^{2}+C_{p_{2}}^{2}-2(\rho_{yp_{1}}C_{y}C_{p_{1}}-\rho_{p_{1}p_{2}}C_{p_{1}}C_{p_{2}}+\rho_{yp_{2}}C_{y}C_{p_{2}})].$$ (22)

Similarly the bias and MSE of ${\bar{y}}_{SK(DP)}$, are given by:

$$Bias({\bar{y}}_{SK(DP)})≅\lambda \bar{Y}[\rho_{p_{1}p_{2}}C_{p_{1}}C_{p_{2}}+\rho_{yp_{1}}C_{y}C_{p_{1}}+\rho_{yp_{2}}C_{y}C_{p_{2}}],$$ (23)

and

$$MSE({\bar{y}}_{SK(DP)})≅{\bar{Y}}^{2}\lambda [C_{y}^{2}+C_{p_{1}}^{2}+C_{p_{2}}^{2}+2(\rho_{yp_{1}}C_{y}C_{p_{1}}+\rho_{p_{1}p_{2}}C_{p_{1}}C_{p_{2}}+\rho_{yp_{2}}C_{y}C_{p_{2}})].$$ (24)

## Proposed class of estimators

In the lines of Shukla et al. [12], we proposed a generalized class of factor type estimators for mean estimator. The proposed estimator, is given by:

$${\bar{y}}_{prop}=\bar{y}[exp(\frac{S_{1}-M_{1}}{S_{1}+M_{1}})exp(\frac{S_{2}-M_{2}}{S_{2}+M_{2}})],$$ (25)

where,

$$S_{1}=(A_{1}+C_{1})P_{1}+fB_{1}p_{1},\;S_{2}=(A_{2}+C_{2})P_{2}+fB_{2}p_{2},\;M_{1}=(A_{1}+fB_{1})P_{1}+C_{1}p_{1},$$

$$M_{2}=(A_{2}+fB_{2})P_{2}+C_{2}p_{2},\;A_{i}=(K_{i}-1)(K_{i}-2),\;B_{i}=(K_{i}-1)(K_{i}-4)\;and$$

$$C_{i}=(K_{i}-2)(K_{i}-3)(K_{i}-4).$$

Substituting different values of *K*_*i*_ (i = 1,2,3,4) in (25), we can generate many more different types of estimators from our general proposed class of estimators (see Table 1).

**Table 1:** Family members of proposed class of estimators.

*S*.*No*.	*K*_1_	*K*_2_	Estimators
1	1	1	${\bar{y}}_{prop\;1}=\bar{y}exp(\frac{P_{1}-p_{1}}{P_{1}+p_{1}})exp(\frac{P_{2}-p_{2}}{P_{2}+p_{2}})$
2	1	2	${\bar{y}}_{prop\;2}=\bar{y}exp(\frac{P_{1}-p_{1}}{P_{1}+p_{1}})exp(\frac{p_{2}-P_{2}}{p_{2}+P_{2}})$
3	1	3	${\bar{y}}_{prop\;3}=\bar{y}exp(\frac{P_{1}-p_{1}}{P_{1}+p_{1}})exp(\frac{n(P_{2}-p_{2})}{2NP_{2}-n(p_{2}+P_{2})})$
4	1	4	${\bar{y}}_{prop\;4}=\bar{y}exp(\frac{P_{1}-p_{1}}{P_{1}+p_{1}})$
5	2	1	${\bar{y}}_{prop\;5}=\bar{y}exp(\frac{p_{1}-P_{1}}{p_{1}+P_{1}})exp(\frac{P_{2}-p_{2}}{P_{2}+p_{2}})$
6	2	2	${\bar{y}}_{prop\;6}=\bar{y}exp(\frac{p_{1}-P_{1}}{p_{1}+P_{1}})exp(\frac{p_{2}-P_{2}}{p_{2}+P_{2}})$
7	2	3	${\bar{y}}_{prop\;7}=\bar{y}exp(\frac{p_{1}-P_{1}}{p_{1}+P_{1}})exp(\frac{n(P_{2}-p_{2})}{2NP_{2}-n(p_{2}+P_{2})})$
8	2	4	${\bar{y}}_{prop\;8}=\bar{y}exp(\frac{p_{1}-P_{1}}{p_{1}+P_{1}})$
9	3	1	${\bar{y}}_{prop\;9}=\bar{y}exp(\frac{n(P_{1}-p_{1})}{2NP_{1}-n(p_{1}+P_{1})})exp(\frac{P_{2}-p_{2}}{P_{2}+p_{2}})$
10	3	2	${\bar{y}}_{prop\;10}=\bar{y}exp(\frac{n(P_{1}-p_{1})}{2NP_{1}-n(p_{1}+P_{1})})exp(\frac{p_{2}-P_{2}}{p_{2}+P_{2}})$
11	3	3	${\bar{y}}_{prop\;11}=\bar{y}exp(\frac{n(P_{1}-p_{1})}{2NP_{1}-n(p_{1}+P_{1})})exp(\frac{n(P_{2}-p_{2})}{2NP_{2}-n(p_{2}+P_{2})})$
12	3	4	${\bar{y}}_{prop\;12}=\bar{y}exp(\frac{n(P_{1}-p_{1})}{2NP_{1}-n(p_{1}+P_{1})})$
13	4	1	${\bar{y}}_{prop\;13}=\bar{y}exp(\frac{P_{2}-p_{2}}{P_{2}+p_{2}})$
14	4	2	${\bar{y}}_{prop\;14}=\bar{y}exp(\frac{p_{2}-P_{2}}{p_{2}+P_{2}})$
15	4	3	${\bar{y}}_{prop\;15}=\bar{y}exp(\frac{n(P_{2}-p_{2})}{2NP_{2}-n(p_{2}+P_{2})})$
16	4	4	${\bar{y}}_{prop\;16}=\bar{y}$

Solving ${\bar{y}}_{prop}$ given in Eq (25) in terms of errors, we have

$${\bar{y}}_{prop}≅\bar{Y}(1+e_{0})[(1+\frac{1}{2}\sigma_{1}e_{1}-\frac{1}{4}\sigma_{1}v_{1}e_{1}^{2}+\frac{1}{8}\sigma_{1}^{2}e_{1}^{2}+\ldots )(1+\frac{1}{2}\sigma_{2}e_{2}-\frac{1}{4}\sigma_{2}v_{2}e_{2}^{2}+\frac{1}{8}\sigma_{2}^{2}e_{2}^{2}+\ldots )],$$ (26)

where

$$\sigma_{1}=\frac{fB_{1}-C_{1}}{A_{1}+fB_{1}+C_{1}},\;v_{1}=\frac{fB_{1}+C_{1}}{A_{1}+fB_{1}+C_{1}},$$

and

$$\sigma_{2}=\frac{fB_{2}-C_{2}}{A_{2}+fB_{2}+C_{2}},\;v_{2}=\frac{fB_{2}+C_{2}}{A_{2}+fB_{2}+C_{2}}.$$

To first order approximation, we have

$$({\bar{y}}_{prop}-\bar{Y})=\bar{Y}[\begin{array}{l} e_{0}+\frac{1}{2}\sigma_{1}e_{1}+\frac{1}{2}\sigma_{2}e_{2}+\frac{1}{2}\sigma_{1}e_{0}e_{1}+\frac{1}{2}\sigma_{2}e_{0}e_{2}-\frac{1}{4}\sigma_{1}v_{1}e_{1}^{2}-\frac{1}{4}\sigma_{2}v_{2}e_{2}^{2}\\ +\frac{1}{8}\sigma_{1}^{2}e_{1}^{2}+\frac{1}{8}\sigma_{2}^{2}e_{2}^{2} \end{array}].$$ (27)

Using (27), the bias and MSE of ${\bar{y}}_{prop}$ are given by:

$$Bias({\bar{y}}_{prop})≅\bar{Y}\lambda [\frac{1}{2}\sigma_{1}\rho_{yp_{1}}C_{y}C_{p_{1}}+\frac{1}{2}\sigma_{2}\rho_{yp_{2}}C_{y}C_{p_{2}}+\frac{1}{8}C_{p_{1}}^{2}(\sigma_{1}^{2}-2\sigma_{1}v_{1})+\frac{1}{8}C_{p_{2}}^{2}(\sigma_{2}^{2}-2\sigma_{2}v_{2})],$$ (28)

and

$$MSE({\bar{y}}_{prop})=\begin{array}{ll} & {\bar{Y}}^{2}\lambda [C_{y}^{2}+\frac{1}{4}\sigma_{1}^{2}C_{p_{1}}^{2}+\frac{1}{4}\sigma_{2}^{2}C_{p_{2}}^{2}+\sigma_{1}\rho_{yp_{1}}C_{y}C_{p_{1}}+\sigma_{2}\rho_{yp_{2}}C_{y}C_{p_{2}}\\ & +\frac{1}{2}\sigma_{1}\sigma_{2}\rho_{p_{1}p_{2}}C_{p_{1}}C_{p_{2}}] \end{array}$$ (29)

Differentiate Eq (29) with respect to *σ*_1_ and *σ*_2_, we get the optimum values of *σ*_1_ and *σ*_2_ i.e.

$$\sigma_{1(opt)}=\frac{2C_{y}(\rho_{yp_{1}}-\rho_{p_{1}p_{2}}\rho_{yp_{2}})}{C_{p_{1}}(\rho_{p_{1}p_{2}}^{2}-1)},$$

and

$$\sigma_{2(opt)}=\frac{2C_{y}(\rho_{yp_{2}}-\rho_{p_{1}p_{2}}\rho_{yp_{1}})}{C_{p_{2}}(\rho_{p_{1}p_{2}}^{2}-1)}.$$

Substituting the optimum values i.e *σ*_1(*opt*)_ and *σ*_2(*opt*)_ in (29), we get minimum MSE of ${\bar{y}}_{prop}$, is given by:

$$MSE({\bar{y}}_{prop}{)}_{min}≅\lambda {\bar{Y}}^{2}C_{y}^{2}[1-R_{yp_{1}p_{2}}^{2}],$$ (30)

where

$$R_{yp_{1}p_{2}}^{2}=\frac{\rho_{yp_{1}}^{2}+\rho_{yp_{2}}^{2}-2\rho_{yp_{1}}\rho_{yp_{2}}\rho_{p_{1}p_{2}}}{1-\rho_{p_{1}p_{2}}^{2}}$$

is the multiple correlation coefficient of *y* on *p*_1_ and *p*_2_.

Now by putting different values of *K*_*i*_ in Eq (25) some members of the proposed class of estimators can be obtained as:

**1.** For *K*_1_ = 1 and *K*_2_ = 1

$${\bar{y}}_{prop\;1}=\bar{y}exp(\frac{P_{1}-p_{1}}{P_{1}+p_{1}})exp(\frac{P_{2}-p_{2}}{P_{2}+p_{2}})$$

The bias and MSE of ${\bar{y}}_{prop\;1}$, are given by:

$$Bias({\bar{y}}_{prop\;1})≅\lambda \bar{Y}[\frac{3}{8}C_{p_{1}}^{2}+\frac{3}{8}C_{p_{2}}^{2}-\frac{1}{2}\rho_{yp_{1}}C_{y}C_{p_{1}}-\frac{1}{2}\rho_{yp_{2}}C_{y}C_{p_{2}}]$$

and

$$MSE({\bar{y}}_{prop\;1})≅\lambda {\bar{Y}}^{2}[C_{y}^{2}+\frac{1}{4}C_{p_{1}}^{2}+\frac{1}{4}C_{p_{2}}^{2}-\rho_{yp_{1}}C_{y}C_{p_{1}}-\rho_{yp_{2}}C_{y}C_{p_{2}}+\frac{1}{2}\rho_{p_{1}p_{2}}C_{p_{1}}C_{p_{2}}].$$

**2.** For *K*_1_ = 1 and *K*_2_ = 2

$${\bar{y}}_{prop\;2}=\bar{y}exp(\frac{P_{1}-p_{1}}{P_{1}+p_{1}})exp(\frac{p_{2}-P_{2}}{p_{2}+P_{2}})$$

The bias and MSE of ${\bar{y}}_{prop\;2}$, are given by:

$$Bias({\bar{y}}_{prop\;2})≅\lambda \bar{Y}[-\frac{1}{8}C_{p_{1}}^{2}-\frac{1}{8}C_{p_{2}}^{2}-\frac{1}{2}\rho_{yp_{1}}C_{y}C_{p_{1}}]$$

and

$$MSE({\bar{y}}_{prop\;2})≅\lambda {\bar{Y}}^{2}[C_{y}^{2}+\frac{1}{4}C_{p_{1}}^{2}+\frac{1}{4}C_{p_{2}}^{2}-\rho_{yp_{1}}C_{y}C_{p_{1}}+\rho_{yp_{2}}C_{y}C_{p_{2}}-\frac{1}{2}\rho_{p_{1}p_{2}}C_{p_{1}}C_{p_{2}}].$$

**3.** For *K*_1_ = 1 and *K*_2_ = 3

$${\bar{y}}_{prop\;3}=\bar{y}exp(\frac{P_{1}-p_{1}}{P_{1}+p_{1}})exp(\frac{n(P_{2}-p_{2})}{2NP_{2}-n(p_{2}+P_{2})})$$

The bias and MSE of ${\bar{y}}_{prop\;3}$, are given by:

$$Bias({\bar{y}}_{prop\;3})≅\lambda \bar{Y}[\frac{3}{8}C_{p_{1}}^{2}+\frac{1}{8}C_{p_{2}}^{2}-\frac{1}{4}(\frac{f}{1-f}{)}^{2}C_{p_{2}}^{2}-\frac{1}{2}\rho_{yp_{1}}C_{y}C_{p_{1}}-\frac{1}{2}(\frac{f}{1-f})\rho_{yp_{2}}C_{y}C_{p_{2}}]$$

and

$$MSE({\bar{y}}_{prop\;3})≅\lambda {\bar{Y}}^{2}[\begin{array}{l} C_{y}^{2}+\frac{1}{4}C_{p_{1}}^{2}+\frac{1}{4}(\frac{f}{1-f}{)}^{2}C_{p_{2}}^{2}-\rho_{yp_{1}}C_{y}C_{p_{1}}-(\frac{f}{1-f})\rho_{yp_{2}}C_{y}C_{p_{2}}\\ +\frac{1}{2}(\frac{f}{1-f})\rho_{p_{1}p_{2}}C_{p_{1}}C_{p_{2}} \end{array}].$$

**4.** For *K*_1_ = 2 and *K*_2_ = 1

$${\bar{y}}_{prop\;5}=\bar{y}exp(\frac{p_{1}-P_{1}}{p_{1}+P_{1}})exp(\frac{P_{2}-p_{2}}{P_{2}+p_{2}})$$

The bias and MSE of ${\bar{y}}_{prop\;5}$, are given by:

$$Bias({\bar{y}}_{prop\;5})≅\lambda \bar{Y}[-\frac{1}{8}C_{p_{1}}^{2}+\frac{3}{8}C_{p_{1}}^{2}+\frac{1}{2}\rho_{yp_{1}}C_{y}C_{p_{1}}-\frac{1}{2}\rho_{yp_{2}}C_{y}C_{p_{2}}]$$

and

$$MSE({\bar{y}}_{prop\;5})≅\lambda {\bar{Y}}^{2}[C_{y}^{2}+\frac{1}{4}C_{p_{1}}^{2}+\frac{1}{4}C_{p_{2}}^{2}+\rho_{yp_{1}}C_{y}C_{p_{1}}-\rho_{yp_{2}}C_{y}C_{p_{2}}-\frac{1}{2}\rho_{p_{1}p_{2}}C_{p_{1}}C_{p_{2}}].$$

**5.** For *K*_1_ = 2 and *K*_2_ = 2

$${\bar{y}}_{prop\;6}=\bar{y}exp(\frac{p_{1}-P_{1}}{p_{1}+P_{1}})exp(\frac{p_{2}-P_{2}}{p_{2}+P_{2}})$$

The bias and MSE of ${\bar{y}}_{prop\;6}$, are given by:

$$Bias({\bar{y}}_{prop\;6})≅\lambda \bar{Y}[-\frac{1}{8}C_{p_{1}}^{2}-\frac{1}{8}C_{p_{1}}^{2}+\frac{1}{2}\rho_{yp_{1}}C_{y}C_{p_{1}}+\frac{1}{2}\rho_{yp_{2}}C_{y}C_{p_{2}}]$$

and

$$MSE({\bar{y}}_{prop\;6})≅\lambda {\bar{Y}}^{2}[C_{y}^{2}+\frac{1}{4}C_{p_{1}}^{2}+\frac{1}{4}C_{p_{2}}^{2}+\rho_{yp_{1}}C_{y}C_{p_{1}}+\rho_{yp_{2}}C_{y}C_{p_{2}}+\frac{1}{2}\rho_{p_{1}p_{2}}C_{p_{1}}C_{p_{2}}].$$

**6.** For *K*_1_ = 2 and *K*_2_ = 3

$${\bar{y}}_{prop\;7}=\bar{y}exp(\frac{p_{1}-P_{1}}{p_{1}+P_{1}})exp(\frac{n(P_{2}-p_{2})}{2NP_{2}-n(p_{2}+P_{2})})$$

The bias and MSE of ${\bar{y}}_{prop\;7}$, are given by:

$$Bias({\bar{y}}_{prop\;7})≅\lambda \bar{Y}[-\frac{1}{8}C_{p_{1}}^{2}-\frac{1}{8}(\frac{f}{1-f})C_{p_{2}}^{2}+\frac{1}{2}\rho_{yp_{1}}C_{y}C_{p_{1}}-\frac{1}{2}(\frac{f}{1-f})\rho_{yp_{2}}C_{y}C_{p_{2}}]$$

and

$$MSE({\bar{y}}_{prop\;7})≅\lambda {\bar{Y}}^{2}[\begin{array}{l} C_{y}^{2}+\frac{1}{4}C_{p_{1}}^{2}+\frac{1}{4}(\frac{f}{1-f}{)}^{2}C_{p_{2}}^{2}+\rho_{yp_{1}}C_{y}C_{p_{1}}-(\frac{f}{1-f})\rho_{yp_{2}}C_{y}C_{p_{2}}\\ -\frac{1}{2}(\frac{f}{1-f})\rho_{p_{1}p_{2}}C_{p_{1}}C_{p_{2}} \end{array}].$$

**7.** For *K*_1_ = 3 and *K*_2_ = 1

$${\bar{y}}_{prop\;9}=\bar{y}exp(\frac{p_{1}-P_{1}}{p_{1}+P_{1}})exp(\frac{n(P_{2}-p_{2})}{2NP_{2}-n(p_{2}+P_{2})})$$

The bias and MSE of ${\bar{y}}_{prop\;9}$, are given by:

$$Bias({\bar{y}}_{prop\;9})≅\lambda \bar{Y}[-\frac{1}{8}(\frac{f}{1-f}{)}^{2}C_{p_{1}}^{2}+\frac{3}{8}C_{p_{2}}^{2}-\frac{1}{2}(\frac{f}{1-f})\rho_{yp_{1}}C_{y}C_{p_{1}}+\frac{1}{2}\rho_{yp_{2}}C_{y}C_{p_{2}}]$$

and

$$MSE({\bar{y}}_{prop\;9})≅\lambda {\bar{Y}}^{2}[\begin{array}{l} C_{y}^{2}+\frac{1}{4}(\frac{f}{1-f}{)}^{2}C_{p_{1}}^{2}+\frac{1}{4}C_{p_{2}}^{2}-(\frac{f}{1-f})\rho_{yp_{1}}C_{y}C_{p_{1}}-\rho_{yp_{2}}C_{y}C_{p_{2}}\\ +\frac{1}{2}(\frac{f}{1-f})\rho_{p_{1}p_{2}}C_{p_{1}}C_{p_{2}} \end{array}].$$

**8.** For *K*_1_ = 3 and *K*_2_ = 2

$${\bar{y}}_{prop\;10}=\bar{y}exp(\frac{n(P_{1}-p_{1})}{2NP_{1}-n(p_{1}+P_{1})})exp(\frac{p_{2}-P_{2}}{p_{2}+P_{2}})$$

The bias and MSE of ${\bar{y}}_{prop\;10}$, are given by:

$$Bias({\bar{y}}_{prop\;10})≅\lambda \bar{Y}[-\frac{1}{8}(\frac{f}{1-f}{)}^{2}C_{p_{1}}^{2}-\frac{1}{8}C_{p_{2}}^{2}-\frac{1}{2}(\frac{f}{1-f})\rho_{yp_{1}}C_{y}C_{p_{1}}+\frac{1}{2}(\frac{f}{1-f})\rho_{yp_{2}}C_{y}C_{p_{2}}]$$

and

$$MSE({\bar{y}}_{prop\;10})≅\lambda {\bar{Y}}^{2}[\begin{array}{l} C_{y}^{2}+\frac{1}{4}(\frac{f}{1-f}{)}^{2}C_{p_{1}}^{2}+\frac{1}{4}C_{p_{2}}^{2}-(\frac{f}{1-f})\rho_{yp_{1}}C_{y}C_{p_{1}}+\rho_{yp_{2}}C_{y}C_{p_{2}}\\ -\frac{1}{2}(\frac{f}{1-f})\rho_{p_{1}p_{2}}C_{p_{1}}C_{p_{2}} \end{array}].$$

**9.** For *K*_1_ = 3 and *K*_2_ = 3

$${\bar{y}}_{prop\;11}=\bar{y}exp(\frac{n(P_{1}-p_{1})}{2NP_{1}-n(p_{1}+P_{1})})exp(\frac{n(P_{2}-p_{2})}{2NP_{2}-n(p_{2}+P_{2})})$$

The bias and MSE of ${\bar{y}}_{prop\;11}$, are given by:

$$Bias({\bar{y}}_{prop\;11})≅\lambda \bar{Y}[-\frac{1}{8}(\frac{f}{1-f}{)}^{2}C_{p_{1}}^{2}-\frac{1}{8}(\frac{f}{1-f}{)}^{2}C_{p_{2}}^{2}-\frac{1}{2}(\frac{f}{1-f})\rho_{yp_{1}}C_{y}C_{p_{1}}-\frac{1}{2}(\frac{f}{1-f})\rho_{yp_{2}}C_{y}C_{p_{2}}]$$

and

$$MSE({\bar{y}}_{prop\;11})≅\lambda {\bar{Y}}^{2}[\begin{array}{l} C_{y}^{2}+\frac{1}{4}(\frac{f}{1-f}{)}^{2}C_{p_{1}}^{2}+\frac{1}{4}(\frac{f}{1-f}{)}^{2}C_{p_{2}}^{2}-(\frac{f}{1-f})\rho_{yp_{1}}C_{y}C_{p_{1}}\\ -(\frac{f}{1-f})\rho_{yp_{2}}C_{y}C_{p_{2}}+\frac{1}{2}(\frac{f}{1-f}{)}^{2}\rho_{p_{1}p_{2}}C_{p_{1}}C_{p_{2}} \end{array}].$$

**10.** For *K*_1_ = 3 and *K*_2_ = 4

$${\bar{y}}_{prop\;12}=\bar{y}exp(\frac{n(P_{1}-p_{1})}{2NP_{1}-n(p_{1}+P_{1})})$$

The bias and MSE of ${\bar{y}}_{prop\;12}$, are given by:

$$Bias({\bar{y}}_{prop\;12})≅\lambda \bar{Y}[-\frac{1}{8}(\frac{f}{1-f}{)}^{2}C_{p_{1}}^{2}-\frac{1}{2}(\frac{f}{1-f})\rho_{yp_{1}}C_{y}C_{p_{1}}]$$

and

$$MSE({\bar{y}}_{prop\;12})≅\lambda {\bar{Y}}^{2}\left[C_{y}^{2}+\frac{1}{4}(\frac{f}{1-f}{)}^{2}C_{p_{1}}^{2}-(\frac{f}{1-f})\rho_{yp_{1}}C_{y}C_{p_{1}}\right].$$

**11.** For *K*_1_ = 4 and *K*_2_ = 1

$${\bar{y}}_{prop\;13}=\bar{y}exp(\frac{P_{2}-p_{2}}{P_{2}+p_{2}})$$

The bias and MSE of ${\bar{y}}_{prop\;13}$, are given by:

$$Bias({\bar{y}}_{prop\;13})≅\lambda \bar{Y}[\frac{3}{8}C_{p_{2}}^{2}-\frac{1}{2}\rho_{yp_{2}}C_{y}C_{p_{2}}]$$

and

$$MSE({\bar{y}}_{prop\;13})≅\lambda {\bar{Y}}^{2}[C_{y}^{2}+\frac{1}{4}C_{p_{2}}^{2}-\rho_{yp_{1}}C_{y}C_{p_{2}}].$$

**12.** For *K*_1_ = 4 and *K*_2_ = 2

$${\bar{y}}_{prop\;14}=\bar{y}exp(\frac{p_{2}-P_{2}}{p_{2}+P_{2}})$$

The bias and MSE of ${\bar{y}}_{prop\;14}$, are given by:

$$Bias({\bar{y}}_{prop\;14})≅\lambda \bar{Y}[-\frac{1}{8}C_{p_{2}}^{2}+\frac{1}{2}\rho_{yp_{2}}C_{y}C_{p_{2}}]$$

and

$$MSE({\bar{y}}_{prop\;14})≅\lambda {\bar{Y}}^{2}[C_{y}^{2}+\frac{1}{4}C_{p_{2}}^{2}+\rho_{yp_{1}}C_{y}C_{p_{2}}].$$

**13.** For *K*_1_ = 4 and *K*_2_ = 3

$${\bar{y}}_{prop\;15}=\bar{y}exp(\frac{n(P_{2}-p_{2})}{2NP_{2}-n(p_{2}+P_{2})})$$

The bias and MSE of ${\bar{y}}_{prop\;15}$, are given by:

$$Bias({\bar{y}}_{prop\;15})≅\lambda \bar{Y}[-\frac{1}{8}(\frac{f}{1-f}{)}^{2}C_{p_{2}}^{2}-\frac{1}{2}(\frac{f}{1-f})\rho_{yp_{2}}C_{y}C_{p_{2}}]$$

and

$$MSE({\bar{y}}_{prop\;15})≅\lambda {\bar{Y}}^{2}[C_{y}^{2}+\frac{1}{4}(\frac{f}{1-f}{)}^{2}C_{p_{2}}^{2}-(\frac{f}{1-f})\rho_{yp_{2}}C_{y}C_{p_{2}}].$$

## Theoretical comparison

In this section, we compare our proposed generalized exponential type estimator with other estimator is given by:

### Under simple random sampling

**1.** From (2) and (30),

$$MSE({\bar{y}}_{prop}{)}_{min}<MSE({\bar{y}}_{0})\;if$$

$$MSE_{\left({\bar{y}}_{o}\right)}-MSE({\bar{y}}_{prop}{)}_{min}>0\;or\;if$$

$$\lambda {\bar{Y}}^{2}C_{y}^{2}-{\bar{Y}}^{2}\lambda C_{y}^{2}[1-R_{yp_{1}p_{2}}^{2}]>0,\;or\;if$$

$$R_{yp_{1}p_{2}}^{2}>0.$$

**2.** From (6) and (30),

$$MSE({\bar{y}}_{prop}{)}_{min}<MSE({\bar{y}}_{R})\;if$$

$$MSE_{\left({\bar{y}}_{R}\right)}-MSE({\bar{y}}_{prop}{)}_{min}>0\;or\;if$$

$${\bar{Y}}^{2}\lambda [C_{y}^{2}+C_{p_{1}}^{2}-2\rho_{yp_{1}}C_{y}C_{p_{1}}]-{\bar{Y}}^{2}\lambda C_{y}^{2}[1-R_{yp_{1}p_{2}}^{2}]>0,\;or\;if$$

$$[C_{p_{1}}^{2}-2\rho_{yp_{1}}C_{y}C_{p_{1}}+C_{y}^{2}R_{yp_{1}p_{2}}^{2}]>0.$$

**3.** From (8) and (30),

$$MSE({\bar{y}}_{prop}{)}_{min}<MSE({\bar{y}}_{P})\;if$$

$$MSE_{\left({\bar{y}}_{P}\right)}-MSE({\bar{y}}_{prop}{)}_{min}>0\;or\;if$$

$${\bar{Y}}^{2}\lambda [C_{y}^{2}+C_{p_{1}}^{2}+2\rho_{yp_{1}}C_{y}C_{p_{1}}]-{\bar{Y}}^{2}\lambda C_{y}^{2}[1-R_{yp_{1}p_{2}}^{2}]>0,\;or\;if$$

$$[C_{p_{1}}^{2}+2\rho_{yp_{1}}C_{y}C_{p_{1}}+C_{y}^{2}R_{yp_{1}p_{2}}^{2}]>0.$$

**4.** From (12) and (30),

$$MSE({\bar{y}}_{prop}{)}_{min}<MSE({\bar{y}}_{exp(R)})\;if$$

$$MSE_{\left({\bar{y}}_{exp(R)}\right)}-MSE({\bar{y}}_{prop}{)}_{min}>0\;or\;if$$

$${\bar{Y}}^{2}\lambda [C_{y}^{2}+\frac{1}{4}C_{p_{1}}^{2}-\rho_{yp_{1}}C_{y}C_{p_{1}}]-{\bar{Y}}^{2}\lambda C_{y}^{2}[1-R_{yp_{1}p_{2}}^{2}]>0,\;or\;if$$

$$[\frac{1}{4}C_{p_{1}}^{2}-\rho_{yp_{1}}C_{y}C_{p_{1}}+C_{y}^{2}R_{yp_{1}p_{2}}^{2}]>0.$$

**5.** From (14) and (30),

$$MSE({\bar{y}}_{prop}{)}_{min}<MSE({\bar{y}}_{exp(P)})\;if$$

$$MSE_{\left({\bar{y}}_{exp(P)}\right)}-MSE({\bar{y}}_{prop}{)}_{min}>0\;or\;if$$

$${\bar{Y}}^{2}\lambda [C_{y}^{2}+\frac{1}{4}C_{p_{1}}^{2}+\rho_{yp_{1}}C_{y}C_{p_{1}}]-{\bar{Y}}^{2}\lambda C_{y}^{2}[1-R_{yp_{1}p_{2}}^{2}]>0,\;or\;if$$

$$[\frac{1}{4}C_{p_{1}}^{2}+\rho_{yp_{1}}C_{y}C_{p_{1}}+C_{y}^{2}R_{yp_{1}p_{2}}^{2}]>0.$$

**6.** From (17) and (30),

$$MSE({\bar{y}}_{prop}{)}_{min}<MSE({\bar{y}}_{KB(RP)})\;if$$

$$MSE_{\left({\bar{y}}_{KB(RP)}\right)}-MSE({\bar{y}}_{prop}{)}_{min}>0\;or\;if$$

$$\lambda {\bar{Y}}^{2}C_{y}^{2}[1-\rho_{yp_{1}}^{2}]-{\bar{Y}}^{2}\lambda C_{y}^{2}[1-R_{yp_{1}p_{2}}^{2}]>0,\;or\;if$$

$$[R_{yp_{1}p_{2}}^{2}-\rho_{yp_{1}}^{2}]>0.$$

**7.** From (22) and (30),

$$MSE({\bar{y}}_{prop}{)}_{min}<MSE_{\left({\bar{y}}_{SK(R)}\right)}\;if$$

$$MSE_{\left({\bar{y}}_{SK(R)}\right)}-MSE({\bar{y}}_{prop}{)}_{min}>0\;or\;if$$

$${\bar{Y}}^{2}\lambda [C_{p_{1}}^{2}+C_{y}^{2}+C_{p_{2}}^{2}-2(\rho_{yp_{1}}C_{y}C_{p_{1}}-\rho_{p_{1}p_{2}}C_{p_{2}}+\rho_{yp_{2}}C_{y}C_{p_{2}})]-{\bar{Y}}^{2}\lambda C_{y}^{2}[1-R_{yp_{1}p_{2}}^{2}]>0,\;or\;if$$

$$[C_{p_{1}}^{2}+C_{p_{2}}^{2}-2(\rho_{yp_{1}}C_{y}C_{p_{1}}-\rho_{p_{1}p_{2}}C_{p_{1}}C_{p_{2}}+\rho_{yp_{2}}C_{y}C_{p_{2}})+C_{y}^{2}R_{yp_{1}p_{2}}^{2}]>0.$$

**8.** From (24) and (30),

$$MSE({\bar{y}}_{prop}{)}_{min}<MSE_{\left({\bar{y}}_{SK(P)}\right)}\;if$$

$$MSE_{\left({\bar{y}}_{SK(P)}\right)}-MSE({\bar{y}}_{prop}{)}_{min}>0\;or\;if$$

$${\bar{Y}}^{2}\lambda [C_{p_{1}}^{2}+C_{y}^{2}+C_{p_{2}}^{2}+2(\rho_{yp_{1}}C_{y}C_{p_{1}}+\rho_{p_{1}p_{2}}C_{p_{2}}+\rho_{yp2}C_{y}C_{p_{2}})]-{\bar{Y}}^{2}\lambda C_{y}^{2}[1-R_{yp_{1}p_{2}}^{2}]>0,\;or\;if$$

$$[C_{p_{1}}^{2}+C_{p_{2}}^{2}+2(\rho_{yp_{1}}C_{y}C_{p_{1}}+\rho_{p_{1}p_{2}}C_{p_{1}}C_{p_{2}}+\rho_{yp_{2}}C_{y}C_{p_{2}})+C_{y}^{2}R_{yp_{1}p_{2}}^{2}]>0.$$

## Numerical comparison under simple random sampling

To observe the performance of our proposed generalized class of estimators with respect to other considered estimators under simple random sampling, we use the following data sets, which earlier used by many authors in literature.

**Population 1**. [Source: Koyuncu and Kadilar [13]]

Let *y* be the number of teachers, *p*_1_ be the number of students both primary and secondary schools in Turkey in 2007 for 923 districts in six regions which is greater than 11440.5 and *p*_2_ be the number of students both primary and secondary schools in Turkey in 2008 for 923 districts in six regions which is greater than 333.1647. We use the proportional allocation.

$\bar{Y}=436.4345,\;P_{1}=2.6625,\;P_{2}=3.125,\;\rho_{yp_{1}}=0.6904898,\;C_{p_{1}}=1.826732,\;\rho_{p_{1}p_{2}}=0.8465885,\;C_{p_{2}}=1.641621,\;C_{y}=1.718333$ and $\rho_{yp_{2}}=0.652149$.

**Population 2.** [Source: Singh [14]]

Let *y* be the estimated number of fish caught by marine recreational fisherman in year 1995, *p*_1_ be the proportion of fishes caught greater than 1000 in 1993 and *p*_2_ be the proportion of fishes caught greater than 2000 in 1994.

$N=69,\;n=14,\;\bar{Y}=4514.89,\;P_{1}=0.7391304,\;P_{2}=0.5507246,\;C_{p_{1}}=0.5984409,\;\rho_{p_{1}p_{2}}=0.6577519,C_{p_{2}}=0.9098277,\;C_{y}=1.350,\;\rho_{yp_{2}}=0.538047$ and $\rho_{yp_{1}}=0.3966081.$

**Population 3.** [Source: www.pbs.gov.pk]

Let *y* be the tobacco area production in hectares during the year 2009, *p*_1_ be the proportion of farms with tobacco cultivation area greater than 500 hectares during the year 2007 and *p*_2_ be the proportion of farms with tobacco cultivation area greater than 800 hectares during the year 2008 for 47 districts of Pakistan.

$N=47,\;n=10,\;\bar{Y}=1004.447,\;P_{1}=0.4255319,\;P_{2}=0.3829787,\;C_{p_{1}}=1.174456,\;\rho_{p_{1}p_{2}}=0.9153857,\;C_{p_{2}}=1.283018,\;C_{y}=2.341245,\;\rho_{yp_{2}}=0.4661508$ and $\rho_{yp_{1}}=0.4395989.$

**Population 4.** [Source: www.pbs.gov.pk]

Let *y* be the cotton production in hectares during the year 2009, *p*_1_ be the proportion of farms with cotton cultivation area greater than 37 hectares during the year 2007 and *p*_2_ be the proportion of farms with cotton cultivation area greater than 35 hectares during the year 2008 for 52 districts of Pakistan.

$N=52,\;n=11,\;\bar{Y}=50.03846,\;P_{1}=0.3846154,\;P_{2}=0.4423077,\;C_{p_{1}}=1.277252,\;\rho_{p_{1}p_{2}}=0.8877181,\;C_{p_{2}}=1.13384,\;C_{y}=1.421524,\;\rho_{yp_{2}}=0.6935718$ and $\rho_{yp_{1}}=0.7369579.$

We use the following expression to obtain the Percentage Relative Efficiency (PRE):

$$PRE=\frac{MSE({\bar{y}}_{0})}{MSE({\bar{y}}_{i})orMSE({\bar{y}}_{i}{)}_{\left(min\right)}}\times 100,$$

where *i* = 0, *R*, *P*, *exp*_(*R*)_, *exp*_(*P*)_, *KB*_(*RP*)_, *SK*_(*DR*)_, *SK*_(*DP*)_ and ${\bar{y}}_{prop}.$

The results based on data sets (1–4) are given in Table 2. It is observed in Table 2, that the proposed esstimators (${\bar{y}}_{prop}$) is more efficient then its competators in SRS.

**Table 2:** Percentage relative efficiency (PRE) with ${\bar{y}}_{0}$.

Estimator	Population 1	Population 2	Population 3	Population 4
${\bar{y}}_{0}$	100	100	100	100
${\bar{y}}_{R}$	151.0459	118.3598	123.3652	207.0429
${\bar{y}}_{P}$	27.79133	64.59406	59.07796	31.9321
${\bar{y}}_{exp_{\left(R\right)}}$	182.3193	114.5063	118.7098	185.2997
${\bar{y}}_{exp_{\left(P\right)}}$	49.58877	81.63674	77.91625	53.64828
${\bar{y}}_{KB_{\left(RP\right)}}$	191.1228	118.6659	123.9537	218.8696
${\bar{y}}_{SK_{\left(DR\right)}}$	48.82051	103.427	90.63836	77.81008
${\bar{y}}_{SK_{\left(DP\right)}}$	13.37497	32.04538	33.25377	16.268981
${\bar{y}}_{prop}$	**551.1775**	**141.3846**	**127.9299**	**222.428**

Table 2 clearly shows that our generalized proposed class of estimators is better than all existing estimators. The product estimators ${\bar{y}}_{P},\;{\bar{y}}_{exp_{\left(P\right)}}$ and ${\bar{y}}_{SK_{\left(DP\right)}}$ perform poor in all four populations because of negative correlation. It is also observed that ${\bar{y}}_{SK_{\left(DR\right)}}$ also perform poor in populations 1, 3 and 4. From this results, we conclude that the generalized proposed class of estimators is more efficient than other estimators. Some members of the proposed family of estimators based on PRE are given in Table 3.

**Table 3:** Percentage relative efficiency of proposed family estimators.

	Population 1	Population 2	Population 3	Population 4
${\bar{y}}_{prop1}$	171.3685	138.4056	126.9301	215.6989
${\bar{y}}_{prop2}$	103.1149	79.9349	95.5017	107.0865
${\bar{y}}_{prop3}$	102.8836	112.9439	123.0256	204.9024
${\bar{y}}_{prop5}$	83.90536	113.9393	102.3297	86.82563
${\bar{y}}_{prop6}$	30.32412	55.59706	57.4785	34.5501
${\bar{y}}_{prop7}$	30.2428	89.68737	84.3266	60.913388
${\bar{y}}_{prop9}$	83.54702	136.1699	125.2152	189.3723
${\bar{y}}_{prop10}$	30.23099	70.94277	80.54632	68.31584
${\bar{y}}_{prop11}$	32.99369	114.0681	112.8626	141.189
${\bar{y}}_{prop12}$	49.40173	104.3372	105.8206	119.4923
${\bar{y}}_{prop13}$	165.2506	133.1671	122.0072	165.0596
${\bar{y}}_{prop14}$	54.01867	67.74307	75.15793	58.40237
${\bar{y}}_{prop15}$	53.8342	109.283	106.7871	115.8713

Table 3 gives the PRE of the proposed family of estimators in simple random sampling. It is obsessed that the proposed family of estimators perform poorly because of poor correlation between study and auxiliary variables.

## Existing estimators in stratified random sampling

The auxiliary information is used in reduction of MSE of various estimators for estimating different population parameters that is mean, variance, ratio of two population means and variances etc. To increase the precision we divide the population into homogeneous groups with respect to some characteristic of interest. Many Statisticians have used the auxiliary information in the estimation of population parameters in stratified random sampling for improving the efficiency of estimators.

Dalabehera and Sahoo [15] proposed different regression type estimators in stratified random sampling with two auxiliary variables. Later Kadilar and Cingi [16] also proposed ratio type estimators in stratified random sampling to get efficient results by extending the estimators Upadhyaya and Singh [17]. Singh and Kumar [11] used transformed variables in stratified random sampling. Koyuncu and Kadilar [13] also used ratio and product types estimators using two auxiliary variables in stratified sampling.

Let *U* = {*U*_1_, *U*_2_….*U*_*N*_} be a finite population of size *N* and let *y*, *p*_1_ and *p*_2_ respectively be the study and two auxiliary attributes associated with each unit *U*_*j*_ = (*j* = 1,2,…,*N*). Assume that a population is stratified into *L* homogeneous strata with the *h*^th^ stratum containing *N*_*h*_ units, where *h* = 1,2,…,*L* such that $\sum_{h=1}^{L}N_{h}=N$. A simple random sample of size *n*_*h*_ is drawn without replacement from the *h*^th^ stratum such that $\sum_{h=1}^{L}n_{h}=n$. Let $\left(y_{hi},p_{1_{hi}},p_{2_{hi}}\right)$ be the observed values of *y*, *p*_1_ and *p*_2_ on the *i*^th^ unit of the *h*^th^ stratum, where *i* = 1,2,…,*n*_*h*_. Moreover, let ${\bar{y}}_{h}=\sum_{h=1}^{n_{h}}\frac{y_{hi}}{n_{h}},\;{\bar{y}}_{st}=\sum_{h=1}^{L}W_{h}{\bar{y}}_{h}$, and ${\bar{Y}}_{h}=\sum_{h=1}^{N_{h}}\frac{y_{hi}}{N_{h}},\;\bar{Y}={\bar{Y}}_{st}=\sum_{h=1}^{L}W_{h}{\bar{Y}}_{h}$ be the sample and population means of *y* respectively, where $W_{h}=\frac{N_{h}}{N}$ is the known stratum weight.

Let

$$e_{0h}=\frac{{\bar{y}}_{h}-{\bar{Y}}_{h}}{{\bar{Y}}_{h}},\;e_{1h}=\frac{p_{1h}-P_{1h}}{P_{1h}}\;and\;e_{2h}=\frac{p_{2h}-P_{2h}}{P_{2h}},$$

such that *E*(*e*_*ih*_) = 0, (*i* = 0,1,2),

$$1E(e_{0h}^{2})=\lambda_{h}C_{yh}^{2},\;E(e_{1h}^{2})=\lambda_{h}C_{p_{1h}}^{2},\;E(e_{2h}^{2})=\lambda_{h}C_{p_{2h}}^{2},\;E(e_{0h}e_{1h})=\lambda_{h}\rho_{yp_{1h}}C_{yh}C_{p_{1h}},$$

$$E(e_{0h}e_{2h})=\lambda_{h}\rho_{yp_{2h}}C_{yh}C_{p_{2h}}\;and\;E(e_{1h}e_{2h})=\lambda_{h}\rho_{p_{1h}p_{2h}}C_{p_{1h}}C_{p_{2h}}.$$

where,

$$1\lambda_{h}=(\frac{1}{n_{h}}-\frac{1}{N_{h}}),\;f_{h}=\frac{n_{h}}{N_{h}},\;C_{yh}^{2}=\frac{S_{yh}^{2}}{{\bar{Y}}_{h}^{2}},\;C_{p_{1h}}^{2}=\frac{S_{p_{1h}}^{2}}{P_{1h}^{2}},\;C_{p_{2h}}^{2}=\frac{S_{p_{2h}}^{2}}{P_{2h}^{2}},\;S_{yh}^{2}=\frac{1}{Nh-1}\sum_{i=1}^{Nh}(y_{hi}-{\bar{Y}}_{h}{)}^{2},$$

$$S_{p_{1h}}^{2}=\frac{1}{Nh-1}\sum_{i=1}^{Nh}(p_{1hi}-P_{1h}{)}^{2}\;and\;S_{p_{2h}}^{2}=\frac{1}{Nh-1}\sum_{i=1}^{Nh}(p_{2hi}-P_{2h}{)}^{2}.$$

Now we discuss the same existing estimators in stratified random sampling.

**1.** The usual mean per unit estimator and its MSE in stratified random sampling, are given by:

$${\bar{y}}_{st}=\sum_{h=1}^{L}W_{h}{\bar{y}}_{h}$$ (31)

and

$$MSE({\bar{y}}_{st})=\sum_{h=1}^{L}W_{h}^{2}\lambda_{h}{\bar{Y}}_{h}^{2}C_{yh}^{2}.$$ (32)

**2.** The usual ratio estimator under stratified random sampling, is given by:

$${\bar{y}}_{Rh}=\sum_{h=1}^{L}W_{h}{\bar{y}}_{h}\left(\frac{P_{1h}}{p_{1h}}\right)$$ (33)

**3.** The usual product estimator under stratified random sampling, is given by:

$${\bar{y}}_{Ph}=\sum_{h=1}^{L}W_{h}{\bar{y}}_{h}\left(\frac{p_{1h}}{P_{1h}}\right)$$ (34)

$$Bias({\bar{y}}_{Rh})≅\sum_{h=1}^{L}W_{h}\lambda_{h}{\bar{Y}}_{h}[C_{p_{1h}}^{2}-\rho_{y_{h}p_{1h}}C_{yh}C_{p_{1h}}],$$ (35)

and

$$MSE({\bar{y}}_{Rh})≅\sum_{h=1}^{L}W_{h}^{2}\lambda_{h}{\bar{Y}}_{h}^{2}[C_{yh}^{2}+C_{p_{1h}}^{2}-2\rho_{y_{h}p_{1h}}C_{yh}C_{p_{1h}}].$$ (36)

Similarly the bias and MSE of the ${\bar{y}}_{Ph}$, are given by:

$$Bias({\bar{y}}_{Ph})≅\sum_{h=1}^{L}W_{h}\lambda_{h}{\bar{Y}}_{h}[\rho_{y_{h}p_{1h}}C_{yh}C_{p_{1h}}],$$ (37)

and

$$MSE({\bar{y}}_{Ph})≅\sum_{h=1}^{L}W_{h}^{2}\lambda_{h}{\bar{Y}}_{h}^{2}[C_{yh}^{2}+C_{p_{1h}}^{2}+2\rho_{y_{h}p_{1h}}C_{yh}C_{p_{1h}}].$$ (38)

**4.** Bahl and Tuteja [9] estimator in stratified random sampling, are given by:

$${\bar{y}}_{exp(Rh)}=\sum_{h=1}^{L}W_{h}{\bar{y}}_{h}exp[\frac{P_{1h}-p_{1h}}{P_{1h}+p_{1h}}],$$ (39)

and

$${\bar{y}}_{exp(Ph)}=\sum_{h=1}^{L}W_{h}{\bar{y}}_{h}exp[\frac{p_{1h}-P_{1h}}{p_{1h}+P_{1h}}].$$ (40)

The bias and MSE of ${\bar{y}}_{exp(Rh)}$, are given by:

$$Bias({\bar{y}}_{exp(Rh)})≅\sum_{h=1}^{L}W_{h}\lambda_{h}{\bar{Y}}_{h}[\frac{3}{8}C_{p_{1h}}^{2}-\frac{1}{2}\rho_{y_{h}p_{1h}}C_{yh}C_{p_{1h}}],$$ (41)

and

$$MSE({\bar{y}}_{exp(Rh)})≅\sum_{h=1}^{L}W_{h}^{2}\lambda_{h}{\bar{Y}}_{h}^{2}[C_{yh}^{2}+\frac{1}{4}C_{p_{1h}}^{2}-\rho_{y_{h}p_{1h}}C_{yh}C_{p_{1h}}].$$ (42)

Similarly the bias and MSE of ${\bar{y}}_{exp(Ph)}$, are given by:

$$Bias({\bar{y}}_{exp(Ph)})≅\frac{1}{2}\sum_{h=1}^{L}W_{h}\lambda_{h}{\bar{Y}}_{h}[\rho_{y_{h}p_{1h}}C_{yh}C_{p_{1h}}-\frac{1}{4}C_{p_{1}}^{2}],$$ (43)

and

$$MSE({\bar{y}}_{exp(Ph)})≅\sum_{h=1}^{L}W_{h}^{2}\lambda_{h}{\bar{Y}}_{h}^{2}[C_{yh}^{2}+\frac{1}{4}C_{p_{1h}}^{2}+\rho_{y_{h}p_{1h}}C_{yh}C_{p_{1h}}].$$ (44)

**5.** Kumar and Bhougal [10] proposed exponential ratio-product type estimator for the population mean in stratified random sampling, is given by:

$${\bar{y}}_{KB(RP_{h})}=\sum_{h=1}^{L}W_{h}{\bar{y}}_{h}[\alpha_{h}exp(\frac{P_{1h}-p_{1h}}{P_{1h}+p_{1h}})+(1-\alpha_{h})exp(\frac{p_{1h}-P_{1h}}{p_{1h}+P_{1h}})]$$ (45)

where *α*_*h*_ is unknown constant.

The bias and minimum MSE of ${\bar{y}}_{KB(RP_{h})}$, are given by:

$$Bias({\bar{y}}_{KB(RP_{h})})≅\sum_{h=1}^{L}W_{h}\lambda_{h}{\bar{Y}}_{h}[\frac{1}{8}(4\alpha_{h}-1)C_{p_{1h}}^{2}-(\alpha_{h}-\frac{1}{2})\rho_{y_{h}p_{1h}}C_{yh}C_{p_{1h}}],$$ (46)

and

$$MSE({\bar{y}}_{KB(RP_{h})}{)}_{min}=\lambda {\bar{Y}}^{2}C_{yh}^{2}[1-\rho_{y_{h}p_{1h}}^{2}].$$ (47)

The optimum value of *α*_*h*_, is given by:

$$\alpha_{hopt}≅\frac{1}{2}+\rho_{y_{h}p_{1h}}\frac{C_{yh}}{C_{p_{2h}}}.$$

**6.** Singh and Kumar [11] suggested double ratio and product type estimators in stratified random sampling, are given by:

$${\bar{y}}_{SK(DR_{h})}=\sum_{h=1}^{L}W_{h}{\bar{y}}_{h}(\frac{P_{1h}}{p_{1h}})(\frac{P_{2h}}{p_{2h}}),$$ (48)

and

$${\bar{y}}_{SK(DP_{h})}=\sum_{h=1}^{L}W_{h}{\bar{y}}_{h}(\frac{p_{1h}}{P_{1h}})(\frac{p_{2h}}{P_{2h}}).$$ (49)

The bias and MSE of ${\bar{y}}_{SK(DR)}$, are given by:

$$Bias({\bar{y}}_{SK(DR_{h})})≅\sum_{h=1}^{L}W_{h}\lambda_{h}{\bar{Y}}_{h}[\begin{array}{l} C_{p_{1h}}^{2}+C_{p_{2h}}^{2}+\rho_{p_{1h}p_{2h}}C_{p_{1h}}C_{p_{2h}}-\rho_{y_{h}p_{h1}}C_{yh}C_{p_{1h}}\\ -\rho_{y_{h}p_{1h}}C_{yh}C_{p_{2h}} \end{array}],$$ (50)

and

$$MSE({\bar{y}}_{SK(DR_{h})})≅\sum_{h=1}^{L}W_{h}^{2}\lambda_{h}{\bar{Y}}_{h}^{2}\left[\begin{array}{l} C_{yh}^{2}+C_{p_{1h}}^{2}+C_{p_{2h}}^{2}-2(\rho_{y_{h}p_{1h}}C_{yh}C_{p_{1h}}\\ -\rho_{p_{1h}p_{2}}C_{p_{1h}}C_{p_{2h}}+\rho_{y_{h}p_{2h}}C_{yh}C_{p_{2h}}) \end{array}\right].$$ (51)

Similarly the bias and MSE of ${\bar{y}}_{SK(DP_{h})}$, are given by:

$$Bias({\bar{y}}_{SK(DP_{h})})≅\sum_{h=1}^{L}W_{h}\lambda_{h}{\bar{Y}}_{h}[\begin{array}{l} \rho_{p_{1h}p_{2h}}C_{p_{1h}}C_{p_{2h}}+\rho_{y_{h}p_{1h}}C_{yh}C_{p_{1h}}+\rho_{y_{h}p_{1h}}\\ C_{yh}C_{p_{2h}} \end{array}],$$ (52)

and

$$MSE({\bar{y}}_{SK(DP_{h})})≅\sum_{h=1}^{L}W_{h}^{2}\lambda_{h}{\bar{Y}}_{h}^{2}\left[\begin{array}{l} C_{yh}^{2}+C_{p_{1h}}^{2}+C_{p_{2h}}^{2}+2(\rho_{y_{h}p_{1h}}C_{yh}C_{p_{1h}}\\ +\rho_{p_{1h}p_{2h}}C_{p_{1h}}C_{p_{2h}}+\rho_{y_{h}p_{2h}}C_{yh}C_{p_{2h}}) \end{array}\right].$$ (53)

### Proposed class of estimators

In the lines of Shukla et al. [12], we proposed a generalized class of estimators in stratified random sampling, is given by:

$${\bar{y}}_{prop_{h}}=\sum_{h=1}^{L}W_{h}{\bar{y}}_{h}[exp(\frac{S_{1h}-M_{1h}}{S_{1h}+M_{1h}})exp(\frac{S_{2h}-M_{2h}}{S_{2h}+M_{2h}})],$$ (54)

where,

$$1S_{1h}=(A_{1h}+C_{1h})P_{1h}+f_{h}B_{1h}p_{1h},\;S_{2h}=(A_{2h}+C_{2h})P_{2h}+f_{h}B_{2h}p_{2h},$$

$$M_{1h}=(A_{1h}+f_{h}B_{1h})P_{1h}+C_{1h}p_{1h},\;M_{2h}=(A_{2h}+f_{h}B_{2h})P_{2h}+C_{2h}p_{2h},$$

$$A_{ih}=(K_{ih}-1)(K_{ih}-2),\;B_{ih}=(K_{ih}-1)(K_{ih}-4)\;and$$

$$C_{ih}=(K_{ih}-2)(K_{ih}-3)(K_{ih}-4).$$

Substituting different values of *K*_*ih*_ (i = 1,2,3,4) in (54), we can generate many more different types of estimators from our general proposed class of estimators, given in Table 4.

**Table 4:** Some members of the proposed class of family of estimators ${\bar{y}}_{prop_{h}}$.

*S*.*No*	*K*_1*h*_	*K*_2*h*_	Estimators
1	1	1	${\bar{y}}_{prop\;1h}=\sum_{h=1}^{L}W_{h}{\bar{y}}_{h}exp(\frac{P_{1h}-p_{1h}}{P_{1h}+p_{1h}})exp(\frac{P_{2h}-p_{2h}}{P_{2h}+p_{2h}})$
2	1	2	${\bar{y}}_{prop\;2h}=\sum_{h=1}^{L}W_{h}{\bar{y}}_{h}exp(\frac{P_{1h}-p_{1h}}{P_{1h}+p_{1h}})exp(\frac{p_{2h}-P_{2h}}{p_{2h}+P_{2h}})$
3	1	3	${\bar{y}}_{prop\;3h}=\sum_{h=1}^{L}W_{h}{\bar{y}}_{h}exp(\frac{P_{1h}-p_{1h}}{P_{1h}+p_{1h}})exp(\frac{n(P_{2h}-p_{2h})}{2NP_{2h}-n(p_{2h}+P_{2h})})$
4	1	4	${\bar{y}}_{prop\;4h}=\sum_{h=1}^{L}W_{h}{\bar{y}}_{h}exp(\frac{P_{1h}-p_{1h}}{P_{1h}+p_{1h}})$
5	2	1	${\bar{y}}_{prop\;5h}=\sum_{h=1}^{L}W_{h}{\bar{y}}_{h}exp(\frac{p_{1h}-P_{1h}}{p_{1h}+P_{1h}})exp(\frac{P_{2h}-p_{2h}}{P_{2h}+p_{2h}})$
6	2	2	${\bar{y}}_{prop\;6h}=\sum_{h=1}^{L}W_{h}{\bar{y}}_{h}exp(\frac{p_{1h}-P_{1h}}{p_{1h}+P_{1h}})exp(\frac{p_{2h}-P_{2h}}{p_{2h}+P_{2h}})$
7	2	3	${\bar{y}}_{prop\;7h}=\sum_{h=1}^{L}W_{h}{\bar{y}}_{h}exp(\frac{p_{1h}-P_{1h}}{p_{1h}+P_{1h}})exp(\frac{n(P_{2h}-p_{2h})}{2NP_{2h}-n(p_{2h}+P_{2h})})$
8	2	4	${\bar{y}}_{prop\;8h}=\sum_{h=1}^{L}W_{h}{\bar{y}}_{h}exp(\frac{p_{1h}-P_{1h}}{p_{1h}+P_{1h}})$
9	3	1	${\bar{y}}_{prop\;9h}=\sum_{h=1}^{L}W_{h}{\bar{y}}_{h}exp(\frac{n(P_{1h}-p_{1h})}{2NP_{1h}-(n(p_{1h}+P_{1h})})exp(\frac{P_{2h}-p_{2h}}{P_{2h}+p_{2h}})$
10	3	2	${\bar{y}}_{prop\;10h}=\sum_{h=1}^{L}W_{h}{\bar{y}}_{h}exp(\frac{n(P_{1h}-p_{1h})}{2NP_{1h}-n(p_{1h}+P_{1h})})exp(\frac{p_{2h}-P_{2h}}{p_{2h}+P_{2h}})$
11	3	3	${\bar{y}}_{prop\;11h}=\sum_{h=1}^{L}W_{h}{\bar{y}}_{h}exp(\frac{n(P_{1h}-p_{1h})}{2NP_{1h}-n(p_{1h}+P_{1h})})exp(\frac{n(P_{2h}-p_{2h})}{2NP_{2h}-(n(p_{2h}+P_{2h})})$
12	3	4	${\bar{y}}_{prop\;12h}=\sum_{h=1}^{L}W_{h}{\bar{y}}_{h}exp(\frac{n(P_{1h}-p_{1h})}{2NP_{1h}-n(p_{1h}+P_{1h})})$
13	4	1	${\bar{y}}_{prop\;13h}=\sum_{h=1}^{L}W_{h}{\bar{y}}_{h}exp(\frac{P_{2h}-p_{2h}}{P_{2h}+p_{2h}})$
14	4	2	${\bar{y}}_{prop\;14h}=\sum_{h=1}^{L}W_{h}{\bar{y}}_{h}exp(\frac{p_{2h}-P_{2h}}{p_{2h}+P_{2h}})$
15	4	3	${\bar{y}}_{prop\;15h}=\sum_{h=1}^{L}W_{h}{\bar{y}}_{h}exp(\frac{n(P_{2h}-p_{2h})}{2NP_{2h}-n(p_{2h}+P_{2h})})$
16	4	4	${\bar{y}}_{prop\;16h}=\sum_{h=1}^{L}W_{h}{\bar{y}}_{h}$

Solving ${\bar{y}}_{prop_{h}}$ given in Eq (54) in terms of errors, we have

$${\bar{y}}_{prop_{h}}=\sum_{h=1}^{L}W_{h}{\bar{Y}}_{h}\left[\begin{array}{l} \left(1+e_{0h})(1+\frac{1}{2}\sigma_{1h}e_{1h}-\frac{1}{4}\sigma_{1h}v_{1h}e_{1h}^{2}+\frac{1}{8}\sigma_{1h}^{2}e_{1h}^{2}+\ldots \right)\\ \left(1+\frac{1}{2}\sigma_{2h}e_{2h}-\frac{1}{4}\sigma_{2h}v_{2h}e_{2h}^{2}+\frac{1}{8}\sigma_{2h}^{2}e_{2h}^{2}+\ldots \right) \end{array}\right],$$ (55)

where

$$\sigma_{1h}=\frac{f_{h}B_{1h}-C_{1h}}{A_{1h}+f_{h}B_{1h}+C_{1h}},\;v_{1h}=\frac{f_{h}B_{1h}+C_{1h}}{A_{1h}+f_{h}B_{1h}+C_{1h}},$$

and

$$\sigma_{2h}=\frac{f_{h}B_{2h}-C_{2h}}{A_{2h}+f_{h}B_{2h}+C_{2h}},\;v_{2h}=\frac{f_{h}B_{2h}+C_{2h}}{A_{2h}+f_{h}B_{2h}+C_{2h}}.$$

To first order approximation, we have

$$({\bar{y}}_{prop_{h}}-{\bar{Y}}_{h})≅\sum_{h=1}^{L}W_{h}{\bar{Y}}_{h}\left[\begin{array}{l} e_{0h}+\frac{1}{2}\sigma_{1h}e_{1h}+\frac{1}{2}\sigma_{2h}e_{2h}+\frac{1}{2}\sigma_{1h}e_{0h}e_{1h}+\frac{1}{2}\sigma_{2h}e_{0h}e_{2h}\\ -\frac{1}{4}\sigma_{1h}v_{1h}e_{1h}^{2}-\frac{1}{4}\sigma_{2h}v_{2h}e_{2h}^{2}+\frac{1}{8}\sigma_{1h}^{2}e_{1h}^{2}+\frac{1}{8}\sigma_{2h}^{2}e_{2h}^{2} \end{array}\right].$$ (56)

Taking squaring and expectation of Eq (56) to first order of approximation, we get the bias and MSE:

$$Bias({\bar{y}}_{prop_{h}})≅\sum_{i-1}^{L}W_{h}{\bar{Y}}_{h}\lambda_{h}\left[\begin{array}{l} \frac{1}{2}\sigma_{1h}\rho_{y_{h}p_{1h}}C_{yh}C_{p_{1h}}+\frac{1}{2}\sigma_{2h}\rho_{y_{h}p_{2h}}C_{yh}C_{p_{2h}}\\ +\frac{1}{8}C_{p_{1h}}^{2}(\sigma_{1h}^{2}-2\sigma_{1h}v_{1h})+\frac{1}{8}C_{p_{2h}}^{2}(\sigma_{2h}^{2}-2\sigma_{2h}v_{2h}) \end{array}\right],$$ (57)

and

$$MSE({\bar{y}}_{prop_{h}})≅\sum_{i=1}^{L}W_{h}^{2}{\bar{Y}}_{h}^{2}\lambda_{h}\left[\begin{array}{l} C_{yh}^{2}+\frac{1}{4}\sigma_{1h}^{2}C_{p_{1h}}^{2}+\frac{1}{4}\sigma_{2h}^{2}C_{p_{2h}}^{2}+\sigma_{1h}\rho_{y_{h}p_{1h}}C_{yh}C_{p_{1h}}\\ +\sigma_{2h}\rho_{y_{h}p_{2h}}C_{yh}C_{p_{2h}}+\frac{1}{2}\sigma_{1h}\sigma_{2h}\rho_{p_{1h}p_{2h}}C_{p_{1h}}C_{p_{2h}} \end{array}\right].$$ (58)

Differentiate Eq (58) with respect to *σ*_1*h*_ and *σ*_2*h*_, we get the optimum values of *σ*_1*h*_ and *σ*_2*h*_ i.e.

$$\sigma_{1h(opt)}=\frac{2C_{yh}(\rho_{y_{h}p_{1h}}-\rho_{p_{1h}p_{2h}}\rho_{y_{h}p_{2h}})}{C_{p_{1h}}(\rho_{p_{1h}p_{2h}}^{2}-1)}\;and\;\sigma_{2h(opt)}=\frac{2C_{yh}(\rho_{y_{h}p_{2h}}-\rho_{p_{1h}p_{2h}}\rho_{y_{h}p_{1h}})}{C_{p_{2h}}(\rho_{p_{1h}p_{2h}}^{2}-1)}.$$

Substituting the optimum values of *σ*_1*h*(opt)_ and *σ*_2*h*(opt)_ in Eq (58), we get minimum MSE of ${\bar{y}}_{prop}$ is given by:

$$MSE({\bar{y}}_{prop_{h}}{)}_{min}≅\sum_{i=1}^{L}W_{h}^{2}{\bar{Y}}_{h}^{2}\lambda_{h}C_{yh}^{2}[1-R_{y_{h}p_{1h}p_{2h}}^{2}],$$ (59)

where

$$R_{y_{h}p_{1h}p_{2h}}^{2}=\frac{\rho_{y_{h}p_{1h}}^{2}+\rho_{y_{h}p_{2h}}^{2}-2\rho_{y_{h}p_{1h}}\rho_{y_{h}p_{2h}}\rho_{p_{1h}p_{2h}}}{1-\rho_{p_{1h}p_{2h}}^{2}}$$

is the multiple correlation coefficient of *y*_*h*_ on *p*_1*h*_ and *p*_2*h*_.

Now by putting different values of *K*_*ih*_ in Eq (54), some member of the proposed class of estimators can be obtained as:

**1.** For *K*_1*h*_ = 1 and *K*_2*h*_ = 1

$${\bar{y}}_{prop\;1h}=\sum_{h=1}^{L}W_{h}{\bar{y}}_{h}exp(\frac{P_{1h}-p_{1h}}{P_{1h}+p_{1h}})exp(\frac{P_{2h}-p_{2h}}{P_{2h}+p_{2h}})$$

The bias and MSE of ${\bar{y}}_{prop\;1h}$, are given by:

$$Bias({\bar{y}}_{prop\;1h})≅\sum_{h=1}^{L}W_{h}\lambda_{h}{\bar{Y}}_{h}[\frac{3}{8}C_{p_{1h}}^{2}+\frac{3}{8}C_{p_{2h}}^{2}-\frac{1}{2}\rho_{y_{h}p_{1h}}C_{yh}C_{p_{1h}}-\frac{1}{2}\rho_{y_{h}p_{2h}}C_{yh}C_{p_{2h}}]$$

and

$$MSE({\bar{y}}_{prop\;1h}]≅\sum_{h=1}^{L}W_{h}^{2}\lambda_{h}{\bar{Y}}_{h}^{2}\left[\begin{array}{l} C_{yh}^{2}+\frac{1}{4}C_{p_{1h}}^{2}+\frac{1}{4}C_{p_{2h}}^{2}-\rho_{y_{h}p_{1h}}C_{yh}C_{p_{1h}}-\rho_{y_{h}p_{2h}}C_{yh}C_{p_{2h}}\\ +\frac{1}{2}\rho_{p_{1h}p_{2h}}C_{p_{1h}}C_{p_{2h}} \end{array}\right].$$

**2.** For *K*_1*h*_ = 1 and *K*_2*h*_ = 2

$${\bar{y}}_{prop\;2h}=\sum_{h=1}^{L}W_{h}{\bar{y}}_{h}exp(\frac{P_{1h}-p_{1h}}{P_{1h}+p_{1h}})exp(\frac{p_{2h}-P_{2h}}{p_{2h}+P_{2h}})$$

The bias and MSE of ${\bar{y}}_{prop\;2h}$, are given by:

$$Bias({\bar{y}}_{prop\;2h})≅\sum_{h=1}^{L}W_{h}\lambda_{h}{\bar{Y}}_{h}[-\frac{1}{8}C_{p_{1h}}^{2}-\frac{1}{8}C_{p_{2h}}^{2}-\frac{1}{2}\rho_{y_{h}p_{1h}}C_{yh}C_{p_{1h}}]$$

and

$$MSE({\bar{y}}_{prop\;2h})≅\sum_{h=1}^{L}W_{h}^{2}\lambda_{h}{\bar{Y}}_{h}^{2}\left[\begin{array}{l} C_{yh}^{2}+\frac{1}{4}C_{p_{1h}}^{2}+\frac{1}{4}C_{p_{2h}}^{2}-\rho_{y_{h}p_{1h}}C_{yh}C_{p_{1h}}+\rho_{y_{h}p_{2h}}C_{yh}C_{p_{2h}}\\ -\frac{1}{2}\rho_{p_{1h}p_{2h}}C_{p_{1h}}C_{p_{2h}} \end{array}\right].$$

**3.** For *K*_1*h*_ = 1 and *K*_2*h*_ = 3

$${\bar{y}}_{prop\;3h}=\sum_{h=1}^{L}W_{h}{\bar{y}}_{h}exp(\frac{P_{1h}-p_{1h}}{P_{1h}+p_{1h}})exp(\frac{n(P_{2h}-p_{2h})}{2NP_{2h}-n(p_{2h}+P_{2h})})$$

The bias and MSE of ${\bar{y}}_{prop\;3h}$, are given by:

$$Bias({\bar{y}}_{prop\;3h})≅\sum_{h=1}^{L}W_{h}\lambda_{h}{\bar{Y}}_{h}\left[\begin{array}{l} \frac{3}{8}C_{p_{1h}}^{2}+\frac{1}{8}C_{p_{2h}}^{2}-\frac{1}{4}(\frac{f_{h}}{1-f_{h}}{)}^{2}C_{p_{2h}}^{2}-\frac{1}{2}\rho_{y_{h}p_{1h}}C_{yh}C_{p_{1h}}\\ -\frac{1}{2}(\frac{f_{h}}{1-f_{h}})\rho_{y_{h}p_{2h}}C_{yh}C_{p_{2h}} \end{array}\right]$$

and

$$MSE({\bar{y}}_{prop\;3h})≅\sum_{h=1}^{L}W_{h}^{2}\lambda_{h}{\bar{Y}}_{h}^{2}\left[\begin{array}{l} C_{yh}^{2}+\frac{1}{4}C_{p_{1h}}^{2}+\frac{1}{4}(\frac{f_{h}}{1-f_{h}}{)}^{2}C_{p_{2h}}^{2}-\rho_{y_{h}p_{1h}}C_{yh}C_{p_{1h}}\\ -(\frac{f_{h}}{1-f_{h}})\rho_{y_{h}p_{2h}}C_{yh}C_{p_{2h}}+\frac{1}{2}(\frac{f_{h}}{1-f_{h}})\rho_{p_{1h}p_{2h}}C_{p_{1h}}C_{p_{2h}} \end{array}\right]$$

**4.** For *K*_1*h*_ = 2 and *K*_2*h*_ = 1

$${\bar{y}}_{prop\;5h}=\sum_{h=1}^{L}W_{h}{\bar{y}}_{h}exp(\frac{p_{1h}-P_{1h}}{p_{1h}+P_{1h}})exp(\frac{P_{2h}-p_{2h}}{P_{2h}+p_{2h}})$$

The bias and MSE of ${\bar{y}}_{prop\;5h}$, are given by:

$$Bias({\bar{y}}_{prop\;5h})≅\sum_{h=1}^{L}W_{h}\lambda_{h}{\bar{Y}}_{h}[-\frac{1}{8}C_{p_{1h}}^{2}+\frac{3}{8}C_{p_{1h}}^{2}+\frac{1}{2}\rho_{y_{h}p_{1h}}C_{yh}C_{p_{1h}}-\frac{1}{2}\rho_{y_{h}p_{2h}}C_{yh}C_{p_{2h}}]$$

and

$$MSE({\bar{y}}_{prop\;5h})≅\sum_{h=1}^{L}W_{h}^{2}\lambda_{h}{\bar{Y}}_{h}^{2}\left[\begin{array}{l} C_{yh}^{2}+\frac{1}{4}C_{p_{1h}}^{2}+\frac{1}{4}C_{p_{2h}}^{2}+\rho_{y_{h}p_{1h}}C_{yh}C_{p_{1h}}-\rho_{y_{h}p_{2h}}C_{yh}C_{p_{2h}}\\ -\frac{1}{2}\rho_{p_{1h}p_{2h}}C_{p_{1h}}C_{p_{2h}} \end{array}\right].$$

**5.** For *K*_1*h*_ = 2 and *K*_2*h*_ = 2

$${\bar{y}}_{prop\;6h}=\sum_{h=1}^{L}W_{h}{\bar{y}}_{h}exp(\frac{p_{1h}-P_{1h}}{p_{1h}+P_{1h}})exp(\frac{p_{2h}-P_{2h}}{p_{2h}+P_{2h}})$$

The bias and MSE of ${\bar{y}}_{prop\;6h}$, are given by:

$$Bias({\bar{y}}_{prop\;6h})≅\sum_{h=1}^{L}W_{h}\lambda_{h}{\bar{Y}}_{h}[-\frac{1}{8}C_{p_{1h}}^{2}-\frac{1}{8}C_{p_{1h}}^{2}+\frac{1}{2}\rho_{y_{h}p_{1}}C_{yh}C_{p_{1h}}+\frac{1}{2}\rho_{y_{h}p_{2h}}C_{yh}C_{p_{2h}}]$$

and

$$MSE({\bar{y}}_{prop\;6h})≅\sum_{h=1}^{L}W_{h}^{2}\lambda_{h}{\bar{Y}}_{h}^{2}\left[\begin{array}{l} C_{yh}^{2}+\frac{1}{4}C_{p_{1h}}^{2}+\frac{1}{4}C_{p_{2h}}^{2}+\rho_{y_{h}p_{1h}}C_{yh}C_{p_{1h}}+\rho_{y_{h}p_{2h}}C_{yh}C_{p_{2h}}\\ +\frac{1}{2}\rho_{p_{1h}p_{2h}}C_{p_{1h}}C_{p_{2h}} \end{array}\right].$$

**6.** For *K*_1*h*_ = 2 and *K*_2*h*_ = 3

$${\bar{y}}_{prop\;7h}=\sum_{h=1}^{L}W_{h}{\bar{y}}_{h}exp(\frac{p_{1h}-P_{1h}}{p_{1h}+P_{1h}})exp(\frac{n(P_{2h}-p_{2h})}{2NP_{2h}-n(p_{2h}+P_{2h})})$$

The bias and MSE of ${\bar{y}}_{prop\;7h}$, are given by:

$$Bias({\bar{y}}_{prop\;7h})≅\sum_{h=1}^{L}W_{h}\lambda_{h}{\bar{Y}}_{h}\left[\begin{array}{l} -\frac{1}{8}C_{p_{1h}}^{2}-\frac{1}{8}(\frac{f_{h}}{1-f_{h}})C_{p_{2h}}^{2}+\frac{1}{2}\rho_{y_{h}p_{1}}C_{yh}C_{p_{1h}}\\ -\frac{1}{2}(\frac{f_{h}}{1-f_{h}})\rho_{y_{h}p_{2h}}C_{yh}C_{p_{2h}} \end{array}\right]$$

and

$$MSE({\bar{y}}_{prop\;7h})≅\sum_{h=1}^{L}W_{h}^{2}\lambda_{h}{\bar{Y}}_{h}^{2}[\begin{array}{ll} & C_{yh}^{2}+\frac{1}{4}C_{p_{1h}}^{2}+\frac{1}{4}(\frac{f_{h}}{1-f_{h}}{)}^{2}C_{p_{2h}}^{2}+\rho_{y_{h}p_{1h}}C_{yh}C_{p_{1h}}\\ & -(\frac{f_{h}}{1-f_{h}})\rho_{y_{h}p_{2h}}C_{yh}C_{p_{2h}}-\frac{1}{2}(\frac{f_{h}}{1-f_{h}})\rho_{p_{1h}p_{2h}}C_{p_{1h}}C_{p_{2h}} \end{array}].$$

**7.** For *K*_1*h*_ = 3 and *K*_2*h*_ = 1

$${\bar{y}}_{prop\;9h}=\sum_{h=1}^{L}W_{h}{\bar{y}}_{h}exp(\frac{n(P_{1h}-p_{1h})}{2NP_{1h}-(n(p_{1h}+P_{1h})})exp(\frac{P_{2h}-p_{2h}}{P_{2h}+p_{2h}})$$

The bias and MSE of ${\bar{y}}_{prop\;9h}$, are given by:

$$Bias({\bar{y}}_{prop\;9h})≅\sum_{h=1}^{L}W_{h}\lambda_{h}{\bar{Y}}_{h}\left[\begin{array}{l} -\frac{1}{8}(\frac{f_{h}}{1-f_{h}}{)}^{2}C_{p_{1h}}^{2}+\frac{3}{8}C_{p_{2h}}^{2}-\frac{1}{2}(\frac{f_{h}}{1-f_{h}})\rho_{y_{h}p_{1h}}C_{yh}C_{p_{1h}}\\ +\frac{1}{2}(\frac{f_{h}}{1-f_{h}})\rho_{y_{h}p_{2h}}C_{yh}C_{p_{2h}} \end{array}\right]$$

and

$$MSE({\bar{y}}_{prop\;9h})≅\sum_{h=1}^{L}W_{h}^{2}\lambda_{h}{\bar{Y}}_{h}^{2}[\begin{array}{l} C_{yh}^{2}+\frac{1}{4}(\frac{f_{h}}{1-f_{h}}{)}^{2}C_{p_{1h}}^{2}+\frac{1}{4}C_{p_{2h}}^{2}-(\frac{f_{h}}{1-f_{h}})\rho_{y_{h}p_{1h}}C_{yh}C_{p_{1h}}\\ -\rho_{y_{h}p_{2h}}C_{yh}C_{p_{2h}}+\frac{1}{2}(\frac{f_{h}}{1-f_{h}})\rho_{p_{1h}p_{2h}}C_{p_{1h}}C_{p_{2h}} \end{array}].$$

**8.** For *K*_1*h*_ = 3 and *K*_2*h*_ = 2

$${\bar{y}}_{prop\;10h}=\sum_{h=1}^{L}W_{h}{\bar{y}}_{h}exp(\frac{n(P_{1h}-p_{1h})}{2NP_{1h}-n(p_{1h}+P_{1h})})exp(\frac{p_{2h}-P_{2h}}{p_{2h}+P_{2h}})$$

The bias and MSE of ${\bar{y}}_{prop\;10h}$, are given by:

$$Bias({\bar{y}}_{prop\;10h})≅\sum_{h=1}^{L}W_{h}\lambda_{h}{\bar{Y}}_{h}\left[\begin{array}{l} -\frac{1}{8}(\frac{f_{h}}{1-f_{h}}{)}^{2}C_{p_{1h}}^{2}-\frac{1}{8}C_{p_{2h}}^{2}-\frac{1}{2}(\frac{f_{h}}{1-f_{h}})\rho_{y_{h}p_{1h}}C_{yh}C_{p_{1h}}\\ +\frac{1}{2}\rho_{y_{h}p_{2h}}C_{yh}C_{p_{2h}} \end{array}\right]$$

and

$$MSE({\bar{y}}_{prop\;10h}≅\sum_{h=1}^{L}W_{h}^{2}\lambda_{h}{\bar{Y}}_{h}^{2}[\begin{array}{l} C_{yh}^{2}+\frac{1}{4}(\frac{f_{h}}{1-f_{h}}{)}^{2}C_{p_{1h}}^{2}+\frac{1}{4}C_{p_{2h}}^{2}-(\frac{f_{h}}{1-f_{h}})\rho_{y_{h}p_{1h}}C_{yh}C_{p_{1h}}\\ +\rho_{y_{h}p_{2h}}C_{yh}C_{p_{2h}}-\frac{1}{2}(\frac{f_{h}}{1-f_{h}})\rho_{p_{1h}p_{2h}}C_{p_{1h}}C_{p_{2h}} \end{array}].$$

**9.** For *K*_1*h*_ = 3 and *K*_2*h*_ = 3

$${\bar{y}}_{prop\;11h}=\sum_{h=1}^{L}W_{h}{\bar{y}}_{h}exp(\frac{n(P_{1h}-p_{1h})}{2NP_{1h}-n(p_{1h}+P_{1h})})exp(\frac{n(P_{2h}-p_{2h})}{2NP_{2h}-(n(p_{2h}+P_{2h})})$$

The bias and MSE of ${\bar{y}}_{prop\;11h}$, are given by:

$$Bias({\bar{y}}_{prop\;11h})≅\sum_{h=1}^{L}W_{h}\lambda_{h}{\bar{Y}}_{h}\left[\begin{array}{l} -\frac{1}{8}(\frac{f_{h}}{1-f_{h}}{)}^{2}C_{p_{1h}}^{2}-\frac{1}{8}(\frac{f_{h}}{1-f_{h}}{)}^{2}C_{p_{2h}}^{2}-\frac{1}{2}(\frac{f_{h}}{1-f_{h}})\rho_{y_{h}p_{1h}}C_{yh}C_{p_{1h}}\\ -\frac{1}{2}(\frac{f_{h}}{1-f_{h}})\rho_{y_{h}p_{2h}}C_{yh}C_{p_{2h}} \end{array}\right]$$

and

$$MSE({\bar{y}}_{prop\;11h})≅\sum_{h=1}^{L}W_{h}^{2}\lambda_{h}{\bar{Y}}_{h}^{2}\left[\begin{array}{l} C_{yh}^{2}+\frac{1}{4}(\frac{f_{h}}{1-f_{h}}{)}^{2}C_{p_{1h}}^{2}+\frac{1}{4}(\frac{f_{h}}{1-f_{h}}{)}^{2}C_{p_{2h}}^{2}-(\frac{f_{h}}{1-f_{h}})\rho_{y_{h}p_{1h}}\\ C_{yh}C_{p_{1h}}-(\frac{f_{h}}{1-f_{h}})\rho_{y_{h}p_{2h}}C_{yh}C_{p_{2h}}+\frac{1}{2}(\frac{f_{h}}{1-f_{h}}{)}^{2}\rho_{p_{1h}p_{2h}}C_{p_{1h}}C_{p_{2h}} \end{array}\right]$$

**10.** For *K*_1*h*_ = 3 and *K*_2*h*_ = 4

$${\bar{y}}_{prop\;12h}=\sum_{h=1}^{L}W_{h}{\bar{y}}_{h}exp(\frac{n(P_{1h}-p_{1h})}{2NP_{1h}-n(p_{1h}+P_{1h})})$$

The bias and MSE of ${\bar{y}}_{prop\;12h}$, are given by:

$$Bias({\bar{y}}_{prop\;12h})≅\sum_{h=1}^{L}W_{h}\lambda_{h}{\bar{Y}}_{h}[-\frac{1}{8}(\frac{f_{h}}{1-f_{h}}{)}^{2}C_{p_{1h}}^{2}-\frac{1}{2}(\frac{f_{h}}{1-f_{h}})\rho_{y_{h}p_{1h}}C_{yh}C_{p_{1h}}]$$

and

$$MSE({\bar{y}}_{prop\;12h})≅\sum_{h=1}^{L}W_{h}^{2}\lambda_{h}{\bar{Y}}_{h}^{2}[C_{yh}^{2}+\frac{1}{4}(\frac{f_{h}}{1-f_{h}}{)}^{2}C_{p_{1h}}^{2}-(\frac{f_{h}}{1-f_{h}})\rho_{y_{h}p_{1h}}C_{yh}C_{p_{1h}}].$$

**11.** For *K*_1*h*_ = 4 and *K*_2*h*_ = 1

$${\bar{y}}_{prop\;13h}=\sum_{h=1}^{L}W_{h}{\bar{y}}_{h}exp(\frac{P_{2h}-p_{2h}}{P_{2h}+p_{2h}})$$

The bias and MSE of ${\bar{y}}_{prop\;13h}$, are given by:

$$Bias({\bar{y}}_{prop\;13h})≅\sum_{h=1}^{L}W_{h}\lambda_{h}{\bar{Y}}_{h}[\frac{3}{8}C_{p_{2h}}^{2}-\frac{1}{2}\rho_{y_{h}p_{2h}}C_{yh}C_{p_{2h}}]$$

and

$$MSE({\bar{y}}_{prop\;13h})≅\sum_{h=1}^{L}W_{h}^{2}\lambda_{h}{\bar{Y}}_{h}^{2}[C_{yh}^{2}+\frac{1}{4}C_{p_{2h}}^{2}-\rho_{y_{h}p_{1h}}C_{yh}C_{p_{2h}}]$$

**12.** For *K*_1*h*_ = 4 and *K*_2*h*_ = 2

$${\bar{y}}_{prop\;14h}=\sum_{h=1}^{L}W_{h}{\bar{y}}_{h}exp(\frac{p_{2h}-P_{2h}}{p_{2h}+P_{2h}})$$

The bias and MSE of ${\bar{y}}_{prop\;14h}$, are given by:

$$Bias({\bar{y}}_{prop\;14})≅\sum_{h=1}^{L}W_{h}\lambda_{h}{\bar{Y}}_{h}[-\frac{1}{8}C_{p_{2h}}^{2}+\frac{1}{2}\rho_{y_{h}p_{2}}C_{yh}C_{p_{2h}}]$$

and

$$MSE({\bar{y}}_{prop\;14h})≅\sum_{h=1}^{L}W_{h}^{2}\lambda_{h}{\bar{Y}}_{h}^{2}[C_{yh}^{2}+\frac{1}{4}C_{p_{2h}}^{2}+\rho_{y_{h}p_{1h}}C_{yh}C_{p_{2h}}].$$

**13.** For *K*_1*h*_ = 4 and *K*_2*h*_ = 3

$${\bar{y}}_{prop\;15h}=\sum_{h=1}^{L}W_{h}{\bar{y}}_{h}exp(\frac{n(P_{2h}-p_{2h})}{2NP_{2h}-n(p_{2h}+P_{2h})})$$

The bias and MSE of ${\bar{y}}_{prop\;15h}$, are given by:

$$Bias({\bar{y}}_{prop\;15h})≅\sum_{h=1}^{L}W_{h}\lambda_{h}{\bar{Y}}_{h}[-\frac{1}{8}(\frac{f_{h}}{1-f_{h}}{)}^{2}C_{p_{2h}}^{2}-\frac{1}{2}(\frac{f_{h}}{1-f_{h}})\rho_{y_{h}p_{2h}}C_{yh}C_{p_{2h}}]$$

and

$$MSE({\bar{y}}_{prop\;15h})≅\sum_{h=1}^{L}W_{h}^{2}\lambda_{h}{\bar{Y}}_{h}^{2}[C_{yh}^{2}+\frac{1}{4}(\frac{f_{h}}{1-f_{h}}{)}^{2}C_{p_{2h}}^{2}-(\frac{f_{h}}{1-f_{h}})\rho_{y_{h}p_{2h}}C_{yh}C_{p_{2h}}].$$

### Theoretical comparison

**1.** From (32) and (59),

$$MSE({\bar{y}}_{prop_{h}}{)}_{min}<MSE({\bar{y}}_{st})\;if$$

$$MSE_{\left({\bar{y}}_{st}\right)}-MSE({\bar{y}}_{prop_{h}}{)}_{min}>0\;or\;if$$

$$\sum_{h=1}^{L}W_{h}^{2}\lambda_{h}{\bar{Y}}_{h}^{2}C_{yh}^{2}-\sum_{i=1}^{L}W_{h}^{2}{\bar{Y}}_{h}^{2}\lambda_{h}C_{yh}^{2}[1-R_{y_{h}p_{1h}p_{2h}}^{2}]>0,orif$$

$$[R_{y_{h}p_{1h}p_{2h}}^{2}]>0$$

**2.** From (36) and (59),

$$MSE({\bar{y}}_{prop}{)}_{min}<MSE({\bar{y}}_{Rh})\;if$$

$$MSE_{\left({\bar{y}}_{Rh}\right)}-MSE({\bar{y}}_{prop}{)}_{min}>0\;or\;if$$

$${\bar{Y}}_{h}^{2}\lambda_{h}[C_{yh}^{2}+C_{p_{1h}}^{2}-2\rho_{y_{h}p_{1h}}C_{yh}C_{p_{1h}}]-{\bar{Y}}_{h}^{2}\lambda C_{yh}^{2}[1-R_{y_{h}p_{1h}p_{2h}}^{2}]>0,orif$$

$$[C_{p_{1h}}^{2}-2\rho_{y_{h}p_{1h}}C_{yh}C_{p_{1h}}+C_{yh}^{2}R_{y_{h}p_{1h}p_{2h}}^{2}]>0$$

**3.** From (38) and (59),

$$MSE({\bar{y}}_{prop}{)}_{min}<MSE({\bar{y}}_{Ph})\;if$$

$$MSE_{\left({\bar{y}}_{Ph}\right)}-MSE({\bar{y}}_{prop}{)}_{min}>0\;or\;if$$

$${\bar{Y}}_{h}^{2}\lambda_{h}[C_{yh}^{2}+C_{p_{1h}}^{2}+2\rho_{y_{h}p_{1h}}C_{yh}C_{p_{1h}}]-{\bar{Y}}_{h}^{2}\lambda C_{yh}^{2}[1-R_{y_{h}p_{1h}p_{2h}}^{2}]>0,orif$$

$$[C_{p_{1h}}^{2}+2\rho_{y_{h}p_{1h}}C_{yh}C_{p_{1h}}+C_{yh}^{2}R_{y_{h}p_{1h}p_{2h}}^{2}]>0$$

**4.** From (42) and (59),

$$MSE({\bar{y}}_{prop}{)}_{min}<MSE({\bar{y}}_{exp(Rh)})\;if$$

$$MSE_{\left({\bar{y}}_{exp(Rh)}\right)}-MSE({\bar{y}}_{prop}{)}_{min}>0\;or\;if$$

$${\bar{Y}}_{h}^{2}\lambda_{h}[C_{yh}^{2}+\frac{1}{4}C_{p_{1h}}^{2}-\rho_{y_{h}p_{1h}}C_{yh}C_{p_{1h}}]-{\bar{Y}}_{h}^{2}\lambda_{h}C_{yh}^{2}[1-R_{y_{h}p_{1h}p_{2h}}^{2}]>0,orif$$

$$[\frac{1}{4}C_{p_{1h}}^{2}-\rho_{y_{h}p_{1h}}C_{yh}C_{p_{1h}}+C_{yh}^{2}R_{y_{h}p_{1h}p_{2h}}^{2}]>0$$

**5.** From (44) and (59),

$$MSE({\bar{y}}_{prop}{)}_{min}<MSE_{\left({\bar{y}}_{exp(Ph)}\right)}\;if$$

$$MSE_{\left({\bar{y}}_{exp(Ph)}\right)}-MSE({\bar{y}}_{prop}{)}_{min}>0\;or\;if$$

$${\bar{Y}}_{h}^{2}\lambda_{h}[C_{y}^{2}+\frac{1}{4}C_{p_{1h}}^{2}+\rho_{y_{h}p_{1h}}C_{yh}C_{p_{1h}}]-{\bar{Y}}_{h}^{2}\lambda C_{yh}^{2}[1-R_{y_{h}p_{1h}p_{2h}}^{2}]>0,orif$$

$$[\frac{1}{4}C_{p_{1h}}^{2}+\rho_{y_{h}p_{1h}}C_{yh}C_{p_{1h}}+C_{yh}^{2}R_{y_{h}p_{1h}p_{2h}}^{2}]>0$$

**6.** From (47) and (59),

$$MSE({\bar{y}}_{prop}{)}_{min}<MSE_{\left({\bar{y}}_{SK(Rh)}\right)}\;if$$

$$MSE_{\left({\bar{y}}_{SK(Rh)}\right)}-MSE({\bar{y}}_{prop}{)}_{min}>0\;or\;if$$

$$\lambda_{h}{\bar{Y}}_{h}^{2}C_{yh}^{2}[1-\rho_{y_{h}p_{1h}}^{2}]-{\bar{Y}}_{h}^{2}\lambda_{h}C_{yh}^{2}[1-R_{y_{h}p_{1h}p_{2h}}^{2}]>0,orif$$

$$[R_{y_{h}p_{1h}p_{2h}}^{2}-\rho_{y_{h}p_{1h}}^{2}]>0$$

**7.** From (51) and (59),

$$MSE({\bar{y}}_{prop}{)}_{min}<MSE_{\left({\bar{y}}_{KB(RP_{h})}\right)}\;if$$

$$MSE_{\left({\bar{y}}_{KB(RP_{h})}\right)}-MSE({\bar{y}}_{prop}{)}_{min}>0\;or\;if$$

$${\bar{Y}}_{h}^{2}\lambda_{h}[C_{p_{1h}}^{2}+C_{yh}^{2}+C_{p_{2h}}^{2}-2(\rho_{y_{h}p_{1h}}C_{yh}C_{p_{1h}}-\rho_{p_{1h}p_{2h}}C_{p_{2h}}+\rho_{y_{h}p_{2h}}C_{yh}C_{p_{2h}})]$$

$$-{\bar{Y}}_{h}^{2}\lambda_{h}C_{y}^{2}[1-R_{y_{h}p_{1h}p_{2h}}^{2}]>0,orif$$

$$\begin{array}{ll} & C_{p_{1h}}^{2}+C_{p_{2h}}^{2}-2(\rho_{y_{h}p_{1h}}C_{yhh}C_{p_{1}}-\rho_{p_{1h}p_{2h}}C_{p_{1h}}C_{p_{2h}}+\rho_{y_{h}p_{2h}}C_{yh}C_{p_{2h}})\\ & +C_{yh}^{2}R_{y_{h}p_{1h}p_{2h}}^{2}>0 \end{array}$$

**8.** From (53) and (58),

$$MSE({\bar{y}}_{prop}{)}_{min}<MSE_{\left({\bar{y}}_{SK(Ph)}\right)}\;if$$

$$MSE_{\left({\bar{y}}_{SK(Ph)}\right)}-MSE({\bar{y}}_{prop}{)}_{min}>0\;or\;if$$

$$\begin{array}{ll} & {\bar{Y}}_{h}^{2}\lambda_{h}[C_{p_{1h}}^{2}+C_{yh}^{2}+C_{p_{2h}}^{2}+2(\rho_{y_{h}p_{1h}}C_{yh}C_{p_{1h}}+\rho_{p_{1h}p_{2h}}C_{p_{1h}}C_{p_{2h}}+\rho_{y_{h}p_{2h}}C_{yh}C_{p_{2h}})]\\ & -{\bar{Y}}_{h}^{2}\lambda_{h}C_{yh}^{2}[1-R_{y_{h}p_{1h}p_{2h}}^{2}]>0,orif \end{array}$$

$$\begin{array}{ll} & C_{p_{1h}}^{2}+C_{p_{2h}}^{2}+2(\rho_{y_{h}p_{1h}}C_{yh}C_{p_{1h}}+\rho_{p_{1h}p_{2h}}C_{p_{2h}}+\rho_{y_{h}p_{2hh}}C_{yh}C_{p_{2h}})\\ & +C_{yh}^{2}R_{y_{h}p_{1h}p_{2h}}^{2}>0 \end{array}$$

## Numerical comparison under stratified random sampling

To observe the performance of our proposed generalized class of estimators with respect to other considered estimators under stratified random sampling, we use the following data sets, which earlier used by many authors.

**Population 1.** [Source: Kadilar and Cingi [16]]

Let *y* be the apple production amount in 1999, *p*_1_ be the proportion of number of apple trees greater than 20,000 in 1999 and *p*_2_ be the proportion of number of apple production amount greater than 25000 in 1998.

In Table 5, the population size is 854 and the sample size is 200 by the use of different strata. Also, find out the coefficient of variation and correlattion coefficeint and conclude that all the correlation is positive among the variables.

**Table 5:** Summary statistics of population 1.

*N*_1_ = 106	*N*_2_ = 106	*N*_3_ = 94	*N*_4_ = 171	*N*_5_ = 204	*N*_6_ = 173
*n*_1_ = 13	*n*_2_ = 24	*n*_3_ = 55	*n*_4_ = 95	*n*_5_ = 10	*n*_6_ = 3
${\bar{Y}}_{1}$ = 1536.774	${\bar{Y}}_{2}=212.594$	${\bar{Y}}_{3}=9384.309$	${\bar{Y}}_{4}=5588.02$	${\bar{Y}}_{5}=966.960$	${\bar{Y}}_{6}=404.39$
$C_{y_{1}}=4.181$	$C_{y_{2}}=5.221$	$C_{y_{3}}=3.187$	$C_{y_{4}}=5.126$	$C_{y_{5}}=2.472$	$C_{y_{6}}=2.339$
$C_{P_{11}}=1.762$	$C_{P_{12}}=1.563$	$C_{P_{13}}=1.095$	$C_{P_{14}}=1.069$	$C_{P_{15}}=1.358$	$C_{P_{16}}=2.773$
$C_{P_{21}}=1.857$	$C_{P_{22}}=1.677$	$C_{P_{23}}=1.220$	$C_{P_{24}}=1.265$	$C_{P_{25}}=1.483$	$C_{P_{26}}=2.942$
$\rho_{y_{1}p_{11}}=0.35$	$\rho_{y_{2}p_{12}}=0.266$	$\rho_{y_{3}p_{13}}=0.332$	$\rho_{y_{4}p_{14}}=0.198$	$\rho_{y_{5}p_{15}}=0.396$	$\rho_{y_{1}p_{16}}=0.673$
$\rho_{y_{1}p_{21}}=0.377$	$\rho_{y_{2}p_{22}}=0.281$	$\rho_{y_{3}p_{23}}=0.363$	$\rho_{y_{4}p_{24}}=0.229$	$\rho_{y_{5}p_{25}}=0.421$	$\rho_{y_{6}p_{26}}=0.677$
$\rho_{p_{11}p_{21}}=0.949$	$\rho_{p_{12}p_{22}}=0.932$	$\rho_{p_{13}p_{23}}=0.897$	$\rho_{p_{14}p_{24}}=0.846$	$\rho_{p_{15}p_{25}}=0.893$	$\rho_{p_{16}p_{26}}=0.883$

**Population 2.** [Source: Sarndal et al. [18]]

Let *y* be the population in thousands during 1985, *p*_1_ be the population proportion during 1975 which is less than 60 and *p*_2_ be the proportion of number of seats in municipal council less than 100. We use proportional allocation.

In Table 6, the population size is 284 and the sample size is 68 by the use of different strata. Also, find out tha sample mean, coefficient of variation and correlattion coefficeint. We conclude that some of the correlation is positive or negative among the variables.

**Table 6:** Summary statistics of population 2.

*N*_1_ = 25	*N*_2_ = 48	*N*_3_ = 32	*N*_4_ = 38	*N*_5_ = 56	*N*_6_ = 41	*N*_7_ = 15	*N*_8_ = 29
*n*_1_ = 6	*n*_2_ = 11	*n*_3_ = 8	*n*_4_ = 9	*n*_5_ = 13	*n*_6_ = 10	*n*_7_ = 4	*n*_8_ = 7
${\bar{Y}}_{1}=62.44$	${\bar{Y}}_{2}=29.60$	${\bar{Y}}_{3}=24.06$	${\bar{Y}}_{4}=31$	${\bar{Y}}_{5}=29.41$	${\bar{Y}}_{6}=20.83$	${\bar{Y}}_{7}=26.67$	${\bar{Y}}_{8}=17.52$
$C_{y_{1}}=1.99$	$C_{y_{2}}=1.22$	$C_{y_{3}}=0.87$	$C_{y_{4}}=1.26$	$C_{y_{5}}=1.92$	$C_{y_{6}}=0.85$	$C_{y_{7}}=0.92$	$C_{y_{8}}=1.24$
$C_{P_{11}}=0.37$	$C_{P_{12}}=0.41$	$C_{P_{13}}=0.26$	$C_{P_{14}}=0.39$	$C_{P_{15}}=0.24$	$C_{P_{16}}=0.22$	$C_{P_{17}}=0.40$	$C_{P_{18}}=0.34$
$C_{P_{21}}=3.46$	$C_{P_{22}}=1.09$	$C_{P_{23}}=1.15$	$C_{P_{24}}=1.32$	$C_{P_{25}}=0.97$	$C_{P_{26}}=1.14$	$C_{P_{27}}=1.10$	$C_{P_{28}}=0.68$
$\rho_{y_{1}p_{11}}=-0.61$	$\rho_{y_{2}p_{12}}=-0.93$	$\rho_{y_{3}p_{13}}=-0.77$	$\rho_{y_{4}p_{14}}=-0.78$	$\rho_{y_{5}p_{15}}=-0.72$	$\rho_{y_{6}p_{16}}=-0.77$	$\rho_{y_{7}p_{17}}=-0.82$	$\rho_{y_{8}p_{18}}=-0.91$
$\rho_{y_{1}p_{21}}=-0.12$	$\rho_{y_{2}p_{22}}=-0.52$	$\rho_{y_{3}p_{23}}=-0.56$	$\rho_{y_{4}p_{24}}=-0.36$	$\rho_{y_{5}p_{25}}=-0.35$	$\rho_{y_{6}p_{26}}=-0.56$	$\rho_{y_{7}p_{27}}=-0.62$	$\rho_{y_{8}p_{28}}=-0.76$
$\rho_{p_{11}p_{21}}=0.10$	$\rho_{p_{12}p_{22}}=0.38$	$\rho_{p_{13}p_{23}}=0.22$	$\rho_{p_{14}p_{24}}=0.29$	$\rho_{p_{15}p_{25}}=0.24$	$\rho_{p_{16}p_{26}}=0.20$	$\rho_{p_{17}p_{27}}=0.36$	$\rho_{p_{18}p_{28}}=0.50$

**Population 3.** [Source: Koyuncu and Kadilar [13]]

Let *y* be the number of teachers, *p*_1_ be the number of students both primary and secondary schools in Turkey in 2007 for 923 districts in six regions which is less then 1000 and *p*_2_ be the number of students both primary and secondary schools in Turkey in 2008 for 923 districts in six regions which is less then 200. We use proportional allocation.

In Table 7, the population size is 923 and the sample size is 180 for different strata. Also, find out tha sample mean, coefficient of variation and correlattion coefficeint. We conclude that all of the correlation is positive among the variables.

**Table 7:** Summary statistics of population 3.

*N*_1_ = 127	*N*_2_ = 117	*N*_3_ = 103	*N*_4_ = 170	*N*_5_ = 205	*N*_6_ = 201
*n*_1_ = 31	*n*_2_ = 21	*n*_3_ = 29	*n*_4_ = 38	*n*_5_ = 22	*n*_6_ = 39
${\bar{Y}}_{1}=703.74$	${\bar{Y}}_{2}=413$	${\bar{Y}}_{3}=573.17$	${\bar{Y}}_{4}=424.67$	${\bar{Y}}_{5}=267.03$	${\bar{Y}}_{6}=393.84$
$C_{y_{1}}=1.25$	$C_{y_{2}}=1.56$	$C_{y_{3}}=1.80$	$C_{y_{4}}=1.90$	$C_{y_{5}}=1.52$	$C_{y_{6}}=1.80$
$C_{P_{11}}=0.35$	$C_{P_{12}}=0.32$	$C_{P_{13}}=0.34$	$C_{P_{14}}=0.52$	$C_{P_{15}}=0.45$	$C_{P_{16}}=0.33$
$C_{P_{21}}=0.82$	$C_{P_{22}}=1.05$	$C_{P_{23}}=0.93$	$C_{P_{24}}=1.26$	$C_{P_{25}}=1.39$	$C_{P_{26}}=0.97$
$\rho_{y_{1}p_{11}}=0.27$	$\rho_{y_{2}p_{12}}=0.19$	$\rho_{y_{3}p_{13}}=0.18$	$\rho_{y_{4}p_{14}}=0.25$	$\rho_{y_{5}p_{15}}=0.25$	$\rho_{y_{6}p_{16}}=0.16$
$\rho_{y_{1}p_{21}}=0.56$	$\rho_{y_{2}p_{22}}=0.48$	$\rho_{y_{3}p_{23}}=0.43$	$\rho_{y_{4}p_{24}}=0.53$	$\rho_{y_{5}p_{25}}=0.58$	$\rho_{y_{6}p_{26}}=0.40$
$\rho_{p_{11}p_{21}}=0.43$	$\rho_{p_{12}p_{22}}=0.30$	$\rho_{p_{13}p_{23}}0.37$	$\rho_{p_{14}p_{24}}=0.41$	$\rho_{p_{15}p_{25}}=0.33$	$\rho_{p_{16}p_{26}}=0.34$

**Population 4.** [Source: Gerard et al. [19]]

Let *y* be the Total Taxation in Euros in 2001, *p*_1_ be the Total Taxable income in Euros in 2001 which less then from mean and *p*_2_ be the Total average income in Euors in 2001 which is less then from mean. We use proportional allocation.

In Table 8, the population size is 589 and the sample size is 150 for different strata. Also, find out tha sample mean, coefficient of variation and correlattion coefficeint. We conclude that all of the correlation is negative among the variables.

**Table 8:** Summary statistics of population 4.

*N*_1_ = 70	*N*_2_ = 111	*N*_3_ = 64	*N*_4_ = 65	*N*_5_ = 69	*N*_6_ = 84	*N*_7_ = 44	*N*_8_ = 44	*N*_9_ = 38
*n*_1_ = 18	*n*_2_ = 28	*n*_3_ = 16	*n*_4_ = 17	*n*_5_ = 18	*n*_6_ = 21	*n*_7_ = 11	*n*_8_ = 11	*n*_9_ = 10
${\bar{Y}}_{1}=81845269$	${\bar{Y}}_{2}=77637833$	${\bar{Y}}_{3}=53163090$	${\bar{Y}}_{4}=72678044$	${\bar{Y}}_{5}=45901248$	${\bar{Y}}_{6}=33367892$	${\bar{Y}}_{7}=52906990$	${\bar{Y}}_{8}=11070725$	${\bar{Y}}_{9}=33313100$
$C_{y_{1}}=2.05$	$C_{y_{2}}=0.88$	$C_{y_{3}}=1.20$	$C_{y_{4}}=1.40$	$C_{y_{5}}=1.35$	$C_{y_{6}}1.68$	$C_{y_{7}}=0.86$	$C_{y_{8}}=0.86$	$C_{y_{9}}=1.63$
$C_{P_{11}}=0.89$	$C_{P_{12}}0.90$	$C_{P_{13}}=0.60$	$C_{P_{14}}=0.93$	$C_{P_{15}}=0.53$	$C_{P_{16}}0.39$	$C_{P_{17}}=0.69$	$C_{P_{18}}=0.15$	$C_{P_{19}}=0.34$
$C_{P_{21}}=0.97$	$C_{P_{22}}=1.72$	$C_{P_{23}}=0.29$	$C_{P_{24}}=1.05$	$C_{P_{25}}=0.31$	$C_{P_{26}}=0.52$	$C_{P_{27}}=0.15$	$C_{P_{28}}=0.40$	$C_{P_{29}}=0.48$
$\rho_{y_{1}p_{11}}=-0.30$	$\rho_{y_{2}p_{12}}=-0.68$	$\rho_{y_{3}p_{13}}=-0.68$	$\rho_{y_{4}p_{14}}=-0.42$	$\rho_{y_{5}p_{15}}=-0.63$	$\rho_{y_{6}p_{16}}=-0.58$	$\rho_{y_{7}p_{17}}=-0.72$	$\rho_{y_{8}p_{18}}=-0.62$	$\rho_{y_{8}p_{19}}=-0.66$
$\rho_{y_{1}p_{21}}=0.10$	$\rho_{y_{2}p_{22}}=0.12$	$\rho_{y_{3}p_{23}}=-0.16$	$\rho_{y_{4}p_{24}}=0.15$	$\rho_{y_{5}p_{25}}=0.037$	$\rho_{y_{6}p_{26}}=0.07$	$\rho_{y_{7}p_{27}}=0.18$	$\rho_{y_{8}p_{28}}=-0.17$	$\rho_{y_{9}p_{29}}=-0.02$
$\rho_{p_{11}p_{21}}=0.11$	$\rho_{p_{12}p_{22}}=-0.18$	$\rho_{p_{13}p_{23}}=0.35$	$\rho_{p_{14}p_{24}}=-0.16$	$\rho_{p_{15}p_{25}}=-0.16$	$\rho_{p_{16}p_{26}}=-0.11$	$\rho_{p_{17}p_{27}}=-0.10$	$\rho_{p_{18}p_{28}}=0.38$	$\rho_{p_{19}p_{29}}=0.05$

We use the following expression to obtain the Percentage Relative Efficiency(PRE):

$$PRE=\frac{MSE({\bar{y}}_{st})}{MSE({\bar{y}}_{ih})orMSE({\bar{y}}_{ih}{)}_{\left(min\right)}}\times 100$$

where i = 0, *Rh*, *Ph*, *exp*_(*Rh*)_, *exp*_(*Ph*)_, $KB_{\left(RP_{h}\right)}SK_{\left(DR_{h}\right)},SK_{\left(DP_{h}\right)}$ and ${\bar{y}}_{prop_{h}}.$

The results based on population 1–4 are given Table 9.

**Table 9:** Percentage relative efficiency (PRE) with respect to ${\bar{y}}_{st}$.

Estimator	Population 1	Population 2	Population 3	Population 4
${\bar{y}}_{st}$	100	100	100	100
${\bar{y}}_{Rh}$	109.3217	74.75077	105.0303	55.80278
${\bar{y}}_{Ph}$	73.55255	132.0828	85.96212	121.2923
${\bar{y}}_{exp_{\left(Rh\right)}}$	108.3332	86.42823	103.9904	75.81773
${\bar{y}}_{exp_{\left(Ph\right)}}$	87.29826	115.3814	93.70092	119.7365
${\bar{y}}_{KB_{\left(RP_{h}\right)}}$	110.3059	189.0565	105.0827	129.4229
${\bar{y}}_{SK_{\left(DR_{h}\right)}}$	92.04641	23.73677	118.2915	46.85948
${\bar{y}}_{SK_{\left(DP_{h}\right)}}$	48.70935	39.4912	42.66078	70.30191
${\bar{y}}_{prop_{h}}$	**112.2599**	**196.8767**	**133.2979**	**131.4789**

The PRE values of different estimators with respect to ${\bar{y}}_{st}$ is given Table 9. Some members of the proposed class of estimators show poor performance because of negative correlation particularly product type estimators. Overall the performance of the proposed estimators ${\bar{y}}_{prop_{h}}$ outperforms as compared to all other considered estimators. The PRE values of some members of the proposed class of estimators are given in Table 10.

**Table 10:** Percentage relative efficiency of proposed family estimator.

	Population 1	Population 2	Population 3	Population 4
${\bar{y}}_{prop\;1h}$	111.015	49.66966	129.2438	72.86185
${\bar{y}}_{prop\;2h}$	97.38008	70.62802	73.5152	65.27101
${\bar{y}}_{prop\;3h}$	110.8836	76.4984	112.1906	76.51009
${\bar{y}}_{prop\;5h}$	101.1683	59.68287	121.5799	108.65973
${\bar{y}}_{prop\;6h}$	72.25059	85.25374	65.48686	98.36143
${\bar{y}}_{prop\;7h}$	95.2416	99.81591	101.7252	119.892
${\bar{y}}_{prop\;9h}$	103.0881	54.31054	126.207	93.1835
${\bar{y}}_{prop\;10h}$	86.46328	78.39059	70.33049	82.51838
${\bar{y}}_{prop\;11h}$	106.4946	83.8941	109.7694	92.26266
${\bar{y}}_{prop\;12h}$	105.0036	95.51832	101.3751	91.55516
${\bar{y}}_{prop\;13h}$	109.8506	54.56871	127.6962	93.51715
${\bar{y}}_{prop\;14h}$	85.38252	77.96911	69.99571	83.52082
${\bar{y}}_{prop\;15h}$	106.2032	87.51429	108.4386	100.6563

From Table 10, we observed that the PRE values of some members of the proposed class of estimators perform poorly. These are expected results because product type estimators are performing poorly.

## Conclusion

In this paper, we proposed an improved class of estimators of finite population mean by utilizing data sets on two auxiliary attributes in both simple random sampling (SRS) and Stratified random sampling (StRS) schemes. Bias and MSE expressions of proposed class of estimators ${\bar{y}}_{prop}$ and ${\bar{y}}_{prop_{h}}$ are acquired upto first order of approximation. It can be seen that, both theoretically and mathematically the proposed class of estimators consistently performs better than the existing estimators considered here under SRS and StRS. Based on these findings, we suggest the utilization of the proposed estimators for efficient estimation of population mean in presence of the auxiliary attributes in SRS and (StRS) schemes, are preferable for future study.
